# Ezetimibe inhibits triple-negative breast cancer proliferation and promotes cell cycle arrest by targeting the PDGFR/AKT pathway

**DOI:** 10.1016/j.heliyon.2023.e21343

**Published:** 2023-10-29

**Authors:** Qinyu He, Lingkai Kong, Weiwei Shi, Ding Ma, Kua Liu, Shuwei Yang, Qilei Xin, Chunping Jiang, Junhua Wu

**Affiliations:** a Jinan Microecological Biomedicine Shandong Laboratory, Shounuo City Light West Block, Qingdao Road 3716#, Huaiyin District, Jinan City, Shandong Province, China; b State Key Laboratory of Pharmaceutical Biotechnology, National Institute of Healthcare Data Science at Nanjing University, Jiangsu Key Laboratory of Molecular Medicine, The Affiliated Drum Tower Hospital of Nanjing University Medical School, Medical School, Nanjing University, 22 Hankou Road, Nanjing, Jiangsu, 210093 China; c Department of Gastroenterology, Third Xiangya Hospital, Central South University, Changsha, Hunan, China

**Keywords:** Breast cancer, Triple-negative breast cancer (TNBC), Ezetimibe, Cell cycle, PDGFRβ, Akt

## Abstract

Cholesterol levels were strongly associated with tumor progression and metastasis. Targeted cholesterol metabolism has broad prospects in tumor treatment. Ezetimibe, the only FDA-approved inhibitor of cholesterol absorption, has been reported to be able to inhibit angiogenesis in liver cancer. However, the efficacy and specific mechanisms of Ezetimibe in the treatment of Triple-Negative Breast Cancer (TNBC)have not been reported. Our research shows Ezetimibe inhibits TNBC cell proliferation and blocks the cell cycle in the G1 phase. Mechanistically, Ezetimibe inhibits the activation of PDGFRβ/AKT pathway, thereby promoting cell cycle arrest and inhibiting cell proliferation. By overexpressing PDGFRβ in TNBC cells, we found that PDGFRβ significantly reduced the inhibitory effect of Ezetimibe on TNBC cell proliferation and the cell cycle. Similarly, SC79, an AKT agonist, can reduce the proliferation inhibitory and cycle-blocking effects of Ezetimibe on TNBC cells. Furthermore, the AKT inhibitor MK2206 enhanced the inhibitory effect of Ezetimibe on the cell cycle and proliferation ability of TNBC cells overexpressing PDGFRβ. In xenograft tumor models, we also found that Ezetimibe inhibited TNBC growth, an effect that can be blocked by overexpression of PDGFR or activation of AKT. In summary, we have demonstrated that EZ inhibits the PDGFR/AKT pathway, thereby halting TNBC cycle progression and tumor growth.

## Highlights

• Ezetimibe blocks the triple-negative breast cancer cell cycle in G1 phase.
• Ezetimibe inhibits the PDGFR/AKT pathway, thereby inhibiting tumor growth.
• In xenograft tumor models, ezetimibe significantly inhibited breast cancer growth.

## 1 Introduction

Breast cancer is currently the most common cancer in terms of incidence and the second leading cause of cancer-related deaths in women [1]. Common treatments for breast cancer include surgical removal, radiation therapy, chemotherapy, and immunotherapy [[2], [3], [4]]. Chemotherapy is an important clinical strategy for treating breast cancer, as it can inhibit or kill tumor cells at different stages of tumor development [5]. However, current chemotherapy drugs for breast cancer not only damage tumor cells but also harm normal cells, often causing drug toxicity, such as weakened physical condition, reduced immunity, and gastrointestinal adverse reactions [6,7], causing great pain to patients and bringing great difficulties to clinical treatment. Therefore, finding a safe and effective breast cancer treatment drug is urgently needed.

Cholesterol is a key component of mammalian cell membranes and can be metabolized into different products in the body or inside cells. Studies have shown that cholesterol and its metabolites promote tumor cell growth, proliferation and migration [[8], [9], [10]], thereby regulating tumor occurrence and development [11]. Tao Zhang et al. found that apolipoprotein A-I (APOA-I) in combination with APOA-I binding protein (AIBP) promotes cholesterol efflux, thereby synergistically inhibiting the growth of intestinal tumors [12]. Liu Wen et al. suggested that when tumor cells are acutely exposed to a high cholesterol environment, the cells exhibit enhanced tumorigenicity and migratory properties [13]. Manran Liu et al. showed that increased cholesterol can activate the PI3K/AKT signal to accelerate the progression of breast cancer [14]. Previous reports have suggested that a high cholesterol environment can promote breast cancer growth in the MMTV-PyMT breast cancer model [15]. In addition, increased cholesterol levels are associated with the recurrence of breast cancer. Some clinical studies have found that breast cancer patients who take cholesterol-lowering drugs (such as statins) have lower recurrence rates and lower progression-free survival rates [16,17]. These studies indicate that cholesterol promotes the occurrence and development of breast cancer.

Currently, drugs that intervene in cholesterol metabolism through different mechanisms have been used for anti-tumor research, and targeting cholesterol storage, absorption, and synthesis may be potential ways to effectively inhibit tumor occurrence and development [18]. The most common drugs that intervene in cholesterol synthesis are statins, which inhibit the action of HMG-CoA reductase in the mevalonic acid pathway [19]. Studies have shown that statins can reduce low-density lipoprotein cholesterol (LDL-C), disrupt cell homeostasis, and affect the activity of cell survival-related proteins, thereby inhibiting tumor growth, such as in prostate cancer, colon cancer, and breast cancer [[19], [20], [21], [22]]. Iizuka-Ohashi Mahiro et al. found that lipophilic statins inhibit the activation of AKT in breast cancer cells and induce cell cycle arrest to inhibit breast cancer progression [22]. PCSK9, which limits the uptake of exogenous lipids, downregulates LDL receptor expression in the liver by binding to LDL receptor/LDL complexes. LDL receptors are degraded as LDL receptor/LDL complexes are internalized and dissociated, leading to increased LDL levels in plasma [23]. Jacome Sanz Dafne et al. found that PF-06446846, a PCSK9-specific inhibitor, reduces LDL-C in plasma and inhibits ovarian cancer cell survival, while increased PCSK9 expression levels in ovarian cancer cells can induce AKT phosphorylation [24]. Acyl-coenzyme A: cholesterol acyltransferase 1 (ACAT1) plays an important role in cholesterol storage in cells and avastatin is a lipid-lowering drug that targets ACAT1. J Li et al. found that avastatin treatment can effectively inhibit the growth and metastasis of pancreatic cancer [25]. These results indicate that drugs that lower cholesterol levels can inhibit tumor progression.

Ezetimibe inhibits the absorption of exogenous cholesterol [26]. Compared with conventional chemotherapy drugs, ezetimibe has the advantages of being safer and having fewer side effects, and its anti-cancer effects have been reported. In prostate cancer, in vivo experiments have shown that ezetimibe can inhibit tumor growth by reducing angiogenesis in mice on a high cholesterol diet [27]. Kristine Pelton et al. showed in the SCID breast cancer model that ezetimibe can stabilize blood vessel structure, suppress angiogenesis to improve the tumor microenvironment, and thus inhibit tumor development [28]. Another study found that ezetimibe can inhibit liver inflammation, fibrosis, and tumor growth in mice with defects in liver-specific phosphatase and tensin homolog (PTEN) under a high-fat diet [29]. This study also demonstrated that ezetimibe inhibits cancer by inhibiting angiogenesis. These studies demonstrate that ezetimibe inhibits tumor growth by reducing angiogenesis in different tumor models, but other mechanisms by which ezetimibe inhibits tumor growth have not been explored.

As mentioned, cholesterol promotes breast cancer growth, and drugs that lower cholesterol, such as lipophilic statins, inhibit breast cancer cell proliferation to inhibit breast cancer growth. Therefore, we are curious whether ezetimibe, also a drug that lowers cholesterol, inhibits breast cancer growth by affecting the cell cycle process, in addition to achieving this effect by reducing angiogenesis. In this study, we intend to observe whether ezetimibe, as a drug that lowers cholesterol, can inhibit the proliferation of TNBC cells by affecting the cell cycle and explore the underlying mechanism.

## 2 Materials and methods

### 2.1 Cell culture

MDA-MB-231 (female human) cells were cultured in DMEM (WISENT, Catalog #319-005-CL) supplemented with 10 % fetal bovine serum (FBS) (WISENT, Catalog #086150035) and 1 % penicillin-streptomycin solution (Gibco, Catalog #15140163). Cultures of 4T1 (mouse) cells contained 10 % FBS and 1 % penicillin-streptomycin solution in DMEM (WISENT, Catalog #319-005-CL). All cells were cultured at 37 °C and 5 % CO2 in an incubator.

### 2.2 Ezetimibe configuration

Ezetimibe solutions for storage and use are prepared by dissolving ezetimibe (MedChemExpress, Catalog #HY-17376) in DMSO. The storage concentration is 100 mM, and the concentration is diluted to the appropriate level when used.

### 2.3 Cell viability assay

Cells were seeded in 96-well plates, allowed to adhere to the plates, and then exposed to various concentrations of ezetimibe for 48 h. Afterward, 10 μL of CCK8 solution was applied to each well and incubated at 37 °C for 1 h. The absorbance for each well was measured using the Molecular Devices ID5 instrument at a wavelength of 450 nm.

### 2.4 Cell cycle

After 48 h of treatment with ezetimibe (ezetimibe concentration: 0, 20, 40 μmol/L), the cells were gently washed twice with sterile PBS phosphate buffer, and an appropriate amount of trypsin solution was added for digestion. A total of 5 × 10^5^ cells were collected by cell counting. The cell suspension was loaded into a sterile centrifuge tube and centrifuged at 1000 rpm for 5 min, and then washed and centrifuged three times to obtain the cell pellet. The relevant reagents were added according to the cell cycle assay kit instructions. The cells were tested by flow cytometry, and finally analyzed with FlowJo software.

### 2.5 Colony formation assay

The cells in logarithmic growth phase were digested with trypsin, resuspended in complete medium to form a cell suspension, and subsequently quantified. Five hundred cells/well were seeded in a 6-well plate culture plate in each experimental group. After the cells adhered, the medium containing DMSO, 20 μmol ezetimibe, and 40 μmol ezetimibe was changed, and the cultures were maintained for a duration of 14 days with regular medium changes every 3 days. After washing once with PBS, 1 mL 4 % paraformaldehyde was added to each well and fixed for 30–60 min. Subsequently, the wells were treated with a solution containing crystal violet stain (1 mL) and incubated for approximately 10–20 min. Cells were washed several times with PBS, allowed to dry, photographed under a microscope, and counted.

### 2.6 Construction of overexpressing PDGFR cell lines

To generate PDGFR-overexpressing cell lines, cells were seeded in 12-well plates at a density of 5 × 10^4^ cells per well. Once the confluence reached 30 %, the cells were infected with lentivirus (GENEChem) (MOI = 10), and the medium was replaced 24 h later. Stable-transformed cell lines were selected using 2 μM puromycin, and PDGFR expression was evaluated by western blotting after one week.

### 2.7 RT-PCR quantitative

Cells were seeded in 12-well plates at a density of 2 × 10^5^ cells per well. Total RNA was extracted using TRIzol reagent (Life Technologies, 15,596–018) and cDNA was synthesized using a reverse transcription kit (Vazyme, R323). Real-time fluorescence quantitative PCR analysis was conducted using the ChamQ SYBR qPCR premix (Vazyme, Q341) under the Applied Biosystems 7500 Real-Time PCR System. The pDGFRβ primers (F: GCAGAAGCCACGCTATG and R: AAGAGTGCGTCCCAGAACAAA) were synthesized by GenScript.

### 2.8 Western blotting analysis

After the cells were washed once with pre-cooled PBS and placed on ice, proteins were extracted by the addition of NP40 lysate. Protein concentration was determined by the BCA method. Equal amounts of protein samples were subjected to polyacrylamide gel electrophoresis and then transferred to polyvinylidene difluoride membranes. The membranes were blocked in 5 % milk for 2 h at room temperature, washed with TBST solution, and then incubated with primary antibodies overnight at 4 °C. After three TBST washes, HRP-conjugated secondary antibody was added and allowed to stand for 1 h at room temperature. The samples were then analyzed using Bio-Rad's ECL chemiluminescence system. Primary antibodies against CDK2(CST, 2546 P, 1:1000), CDK4(CST, 2906 P, 1:1000), Cyclin D1(CST, 2978 S, 1:1000), PDGF receptor β（28E1）(CST, 3169 T, 1: 1000), Akt (CST, 9271 S, 1:1000), Phospho-Akt (Ser 473）(CST, 4060 T, 1:1000), GAPDH(Biogot technology, MB001, 1:1000).

### 2.9 Xenograft model

Five-week-old female BALB/c nude mice were purchased from Yangzhou University for the in vivo tumorigenicity study. Mice were injected subcutaneously with 1 × 10^7^ MDA-MB-231 cells stably expressing PDGFRβ and control cells. Five mice were used for each group. When the tumor reached approximately 50 or 100 mm^3^, the mice were randomly grouped. Tumor diameters were measured every 2 days using a Vernier caliper, and the tumor volume was calculated by formula 0.5 × length × width^2^. When the tumor volume reached 2000 mm^3^, the mice were euthanized. Tumor tissue was collected and photographed, and finally, the tumor was preserved with 4 % paraformaldehyde for immunohistochemistry experiments.

### 2.10 Statistical analysis

GraphPad Prism 8.0 was used to analyze statistics. Differences between the two groups were compared using an independent *t*-test, and a statistically significant difference was defined as P < 0.05.

## 3 Result

### 3.1 Ezetimibe inhibits the proliferation and cell cycle progression of TNBC cells

**Fig. 1:**
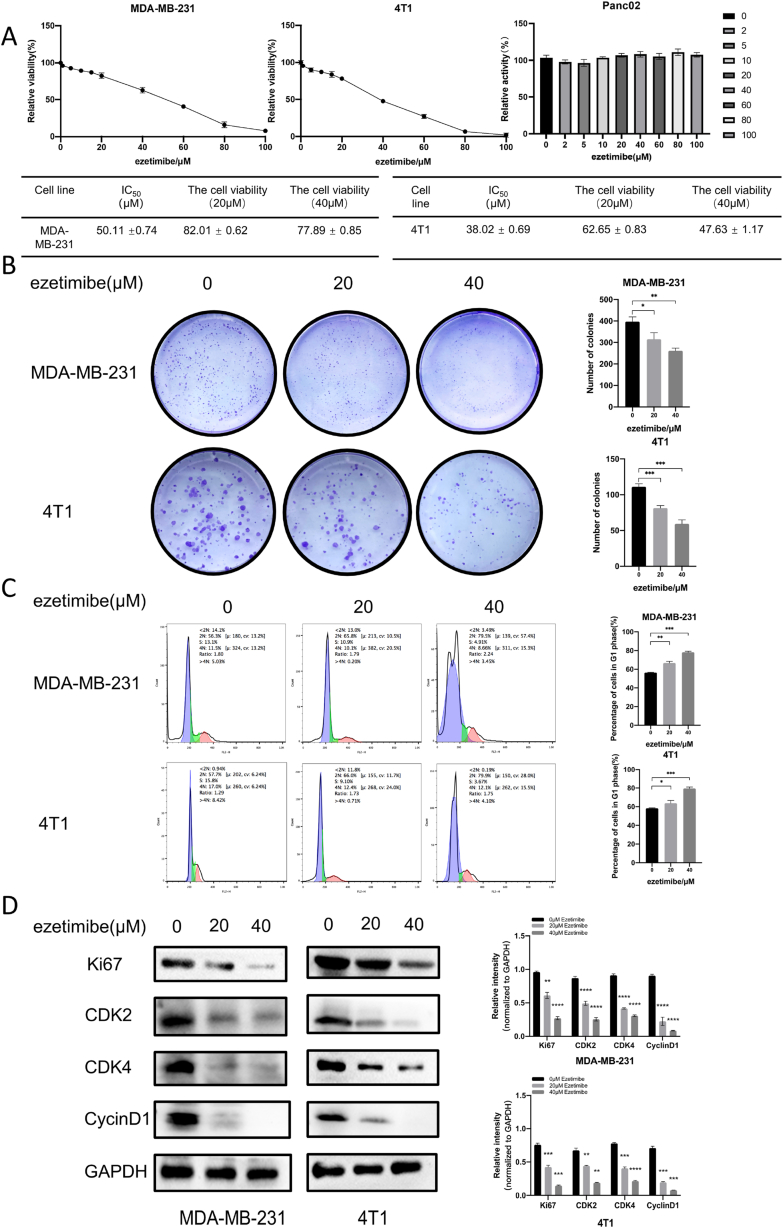
**Effects of Ezetimibe on the viability, proliferation, and cell cycle of TNBC cells.** (A) Viability of MDA-MB-231 and 4T1 cells treated with different concentrations of Ezetimibe (0, 1, 5, 10, 15, 20, 40, 60, 80, 100 μmol/L) for 48 h was detected using the CCK-8 assay. (B) Cell cloning experiments were performed to measure the colony formation of MDA-MB-231 cells after 10 days and 4T1 cells after 14 days of treatment with different concentrations of Ezetimibe (0, 20, 40 μmol/L). (C) Flow cytometry analysis was conducted to examine cell cycle distribution of MDA-MB-231 and 4T1 cells treated with different concentrations of Ezetimibe (0, 20, 40 μmol/L) for 48 h. (D) Western blotting was performed to determine the expression levels of Ki67, CDK2, CDK4, and CyclinD1 proteins in MDA-MB-231 and 4T1 cells treated with different concentrations of Ezetimibe (0, 20, 40 μmol/L) for 48 h. Representative results from three independent experiments are shown, and the data are presented as mean ± standard deviation (Mean ± SD): *p < 0.05, **p < 0.01, ***p < 0.001, ****p < 0.0001. Here 0 μmol/L Ezetimibe refers to 0.1 % (v/v) DMSO solution.

To evaluate the impact of Ezetimibe on the proliferation of TNBC cells, we conducted CCK8 experiments to assess the effects of Ezetimibe on the viability of MDA-MB-231 and 4T1 TNBC cells. The IC_50_ values of Ezetimibe for 48 h of treatment were determined to be 50.11 μM ± 0.74 μM and 38.02 μM ± 0.69 μM for MDA-MB-231 and 4T1 cells, respectively. However, ezetimibe had no effect on PancO2 cells, a pancreatic cancer cell line (Fig. 1A). The cell viabilities of MDA-MB-231 and 4T1 cells after 48 h of treatment with 20 μM Ezetimibe were 82.01 ± 0.62 % and 77.89 ± 0.85 %, respectively (Fig. 1A). Similarly, the cell viabilities of MDA-MB-231 and 4T1 cells treated with 40 μM Ezetimibe were 62.65 ± 0.83 % and 47.63 ± 1.17 %, respectively (Fig. 1A). These results indicate that Ezetimibe at 20 μM and 40 μM significantly affects the cell viability of MDA-MB-231 and 4T1 TNBC cells; hence, 20 μM and 40 μM were selected for studying the effects of Ezetimibe on cell proliferation. Cell cloning experiments showed that the number of colonies formed by MDA-MB-231 and 4T1 cells treated with 20 μM and 40 μM Ezetimibe was significantly lower than that of the control group cells, and the number of colonies showed dose-dependent inhibition (Fig. 1B). Flow cytometry demonstrated that the G1 phase cell population of MDA-MB-231 and 4T1 cells significantly increased after 48 h of Ezetimibe treatment, and this effect was also dose-dependent (Fig. 1C). Western blotting experiments revealed that the levels of the cell proliferation marker Ki67 and the cell cycle markers CDK2, CDK4, and CyclinD1 were significantly reduced in MDA-MB-231 and 4T1 cells treated with Ezetimibe compared to control cells (Fig. 1D). These results indicate that Ezetimibe can significantly inhibit cell proliferation and induce G1/S phase arrest in TNBC cells.

### 3.2 Ezetimibe inhibits PDGFRβ expression in TNBC cells

**Fig. 2:**
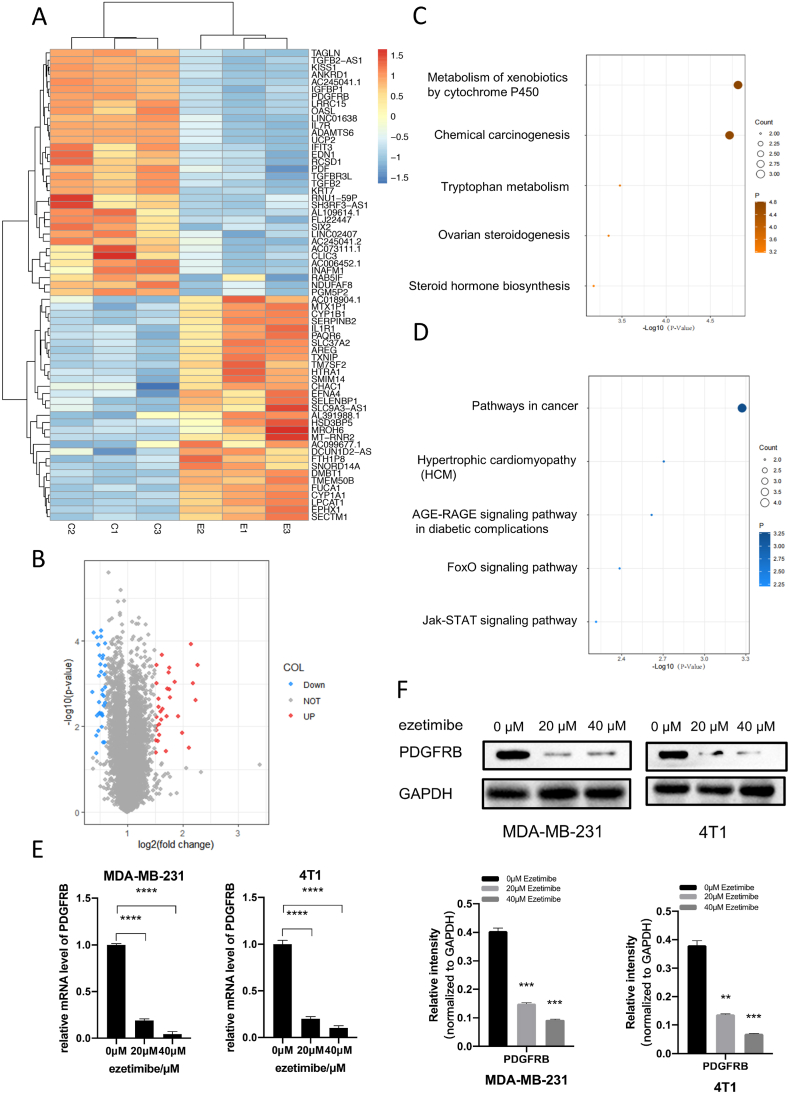
Effects of Ezetimibe on the transcriptome of TNBC cells and its impact on levels of PDGFRβ mRNA and protein. (A) MDA-MB-231 cells were treated with 20 μM Ezetimibe for 48 h, followed by transcriptome sequencing. Genes with fold change >2 for upregulation and <0.6 for downregulation were selected as differentially expressed genes between the 20 μM Ezetimibe treatment group and the control group. (B) Volcano plot of differential genes in(A). (C) and (D) Gene Ontology was utilized to extract and merge enriched pathways, and the distribution of enriched q-values for each pathway was plotted. (C) Represents the pathway enrichment analysis for the upregulated genes in (A), while (D) represents the pathway enrichment analysis for the downregulated genes in (A). (E) MDA-MB-231 and 4T1 cells were treated with different concentrations of Ezetimibe (0, 20, 40 μmol/L) for 48 h, and the mRNA levels of PDGFRβ were measured using qRT-PCR. (F) MDA-MB-231 and 4T1 cells were treated with different concentrations of Ezetimibe (0, 20, 40 μmol/L) for 48 h, and the expression of PDGFRβ protein was assessed using Western blot. The representative results shown in the figure are the mean ± standard deviation (Mean ± SD) of three independent experiments: *p < 0.05, **p < 0.01, ***p < 0.001, ****p < 0.0001. Here 0 μmol/L Ezetimibe refers to 0.1 % (v/v) DMSO solution.

To investigate the mechanism of the effect of Ezetimibe on TNBC cells, we compared the gene expression profiles of Ezetimibe-treated and untreated MDA-MB-231 cells using transcriptome sequencing. Differential gene expression profiles were obtained (see Supplementary Table S1) based on a fold change greater than 2 for upregulated genes and less than 0.6 for downregulated genes, with Fragments per Kilobase Million (FPKM) values greater than 1 and *P*-values less than 0.05 as criteria. A total of 34 downregulated genes and 31 upregulated genes were identified in the Ezetimibe-treated cells based on these criteria (Fig. 2A and B). The hierarchical clustering analysis of differentially expressed genes from the transcriptome sequencing is shown in Fig. 2A. Volcano plot of the differential gene in Fig. 2B. The Gene Ontology (GO) analysis results for differentially expressed genes are shown in Fig. 2C and D. Based on the enrichment level q-value of pathways, we focused on the downregulated genes affected by Ezetimibe. The top three downregulated genes based on fold change were interleukin-7 receptor (IL-7R), platelet-derived growth factor receptor beta (PDGFRβ), and transforming growth factor beta 2 (TGFβ2). Since we are interested in genes related to cell proliferation and the cell cycle, and both PDGFRβ and TGFβ2 are associated with cell proliferation [[30], [31], [32]], we selected PDGFRβ, which showed a higher fold change, for further investigation. qPCR and Western blotting experiments demonstrated a significant decrease in the mRNA and protein expression levels of PDGFRβ in Ezetimibe-treated MDA-MB-231 and 4T1 cells compared to untreated cells (Fig. 2E and F). These results are consistent with the transcriptome sequencing data and suggest that PDGFRβ may play a critical role in the inhibition of TNBC cell proliferation and cell cycle arrest by Ezetimibe.

The inhibitory effect of Ezetimibe on the proliferation of TNBC cells and its ability to block the cell cycle are weakened by the overexpression of PDGFRβ.

**Fig. 3:**
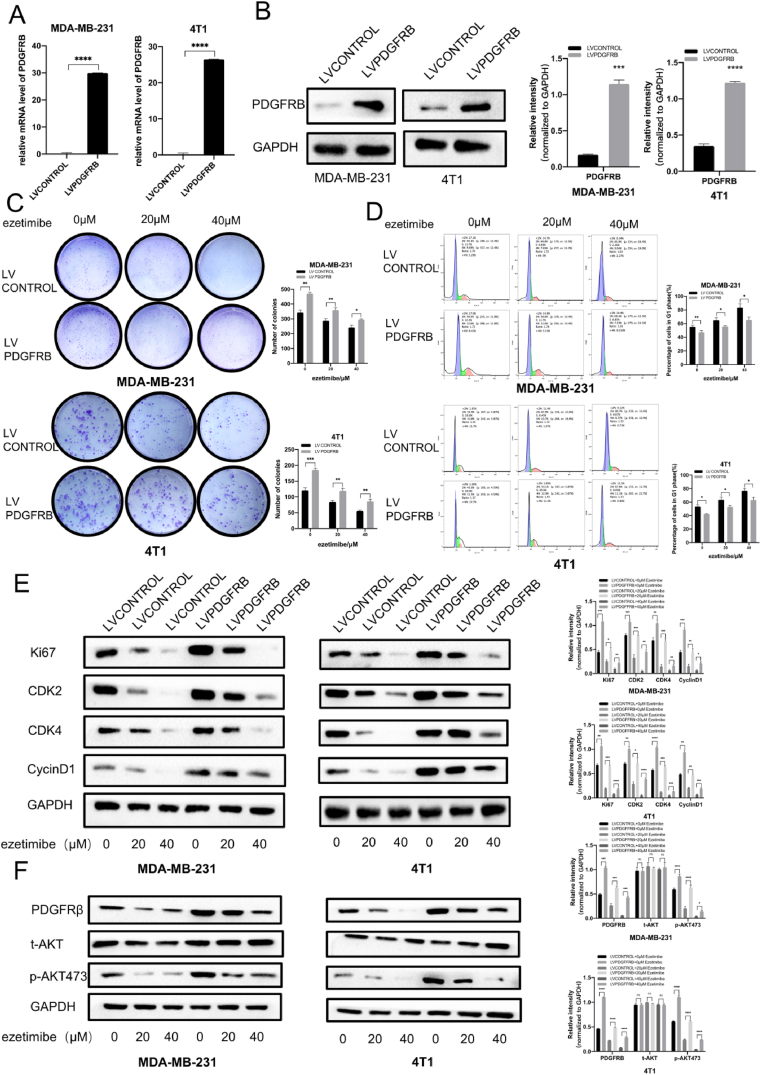
**The impact of PDGFRβ overexpression on the inhibition of MDA-MB-231 and 4T1 cell proliferation and the ability of Ezetimibe to arrest the cell cycle.** (A) (B) qRT-PCR and Western blotting were respectively performed to detect the mRNA and protein levels of PDGFRβ in PDGFRβ-overexpressing MDA-MB-231 and 4T1 cells, respectively, as well as in the control group cells. (C) PDGFRβ-overexpressing or vector-transfected MDA-MB-231 cells were treated with different concentrations of Ezetimibe (0, 20, 40 μmol/L) for 10 days, while PDGFRβ-overexpressing or vector-transfected 4T1 cells were treated for 14 days. The cell clone formation experiment was conducted to determine the number of cell clones formed. (D) PDGFRβ-overexpressing MDA-MB-231 and 4T1 cells, as well as vector-transfected control cells, were treated with different concentrations of Ezetimibe (0, 20, 40 μmol/L) for 48 h, and flow cytometry was used to detect the cell cycle distribution. (E) PDGFRβ-overexpressing MDA-MB-231 and 4T1 cells, as well as vector-transfected control group cells, were treated with different concentrations of Ezetimibe (0, 20, 40 μmol/L) for 48 h. Western blotting was used to examine the expression of Ki67, CDK2, CDK4, and Cyclin D1 proteins. (F) PDGFRβ-overexpressing MDA-MB-231 and 4T1 cells, as well as vector-transfected control group cells, were treated with different concentrations of Ezetimibe (0, 20, 40 μmol/L) for 48 h. Western blotting was performed to analyze the expression of PDGFRβ, *t*-AKT, and *p*-AKT473 proteins. The results presented in the figure are representative of three independent experiments and are expressed as the mean ± standard deviation (Mean ± SD): *p < 0.05, **p < 0.01,***p < 0.001, ****p < 0.0001. Here 0 μmol/L Ezetimibe refers to 0.1 % (v/v) DMSO solution.

We used lentiviral transduction techniques to overexpress PDGFRβ in MDA-MB-231 and 4T1 cells, and qPCR and Western Blotting experiments were used to validate the stable overexpression of PDGFRβ in MDA-MB-231 and 4T1 cells (Fig. 3A and B). After treatment with different concentrations of Ezetimibe, the number of colonies formed by MDA-MB-231 and 4T1 cells overexpressing PDGFRβ was significantly higher than that formed by control cells transfected with empty vector and treated with different concentrations of Ezetimibe (Fig. 3C). These results demonstrate that the overexpression of PDGFRβ in TNBC cells significantly counteracts the inhibitory effect of Ezetimibe on cell proliferation. After treatment with different concentrations of Ezetimibe, the number of cells arrested in the G1 phase was significantly lower in MDA-MB-231 and 4T1 TNBC cells overexpressing PDGFRβ than in control cells transfected with empty vector and treated with different concentrations of Ezetimibe (Fig. 3D). This indicates that overexpression of PDGFRβ in TNBC cells significantly weakens the ability of Ezetimibe to arrest cells in the G1/S phase. Compared to control cells transfected with empty vector and treated with different concentrations of Ezetimibe, the protein levels of Ki67, CDK2, CDK4, and CyclinD1 were significantly higher in MDA-MB-231 and 4T1 TNBC cells overexpressing PDGFRβ and treated with different concentrations of Ezetimibe (Fig. 3E). These results validate that overexpression of PDGFRβ in TNBC cells significantly reverses the inhibitory effects of Ezetimibe on proliferation and cell cycle-related proteins. They also confirmed that overexpression of PDGFRβ in TNBC cells significantly weakened the inhibitory effects of Ezetimibe on cell proliferation and the ability to arrest cells in the G1/S phase.

Studies by Sun et al. have shown that PDGFRβ can activate the PI3K/AKT pathway and promote breast cancer cell proliferation and survival [33,34], suggesting that Ezetimibe may inhibit TNBC growth by regulating the PDGFRβ/AKT signaling pathway. In PDGFRβ-overexpressing TNBC cells without the Ezetimibe treatment, the protein levels of PDGFRβ and *p*-AKT473 significantly increased compared to those in empty vector-transfected control cells without the treatment of Ezetimibe (Fig. 3F). These results suggest that Ezetimibe inhibits the activation of the PDGFRβ/AKT pathway and that PDGFRβ overexpression can reverse this effect, indicating that PDGFRβ is a key target by which ezetimibe inhibits the proliferation of TNBC cells.

AKT activation attenuated the inhibitory effect of Ezetimibe on the proliferation of TNBC cells and the cell cycle arrest of TNBC cells.

**Fig. 4:**
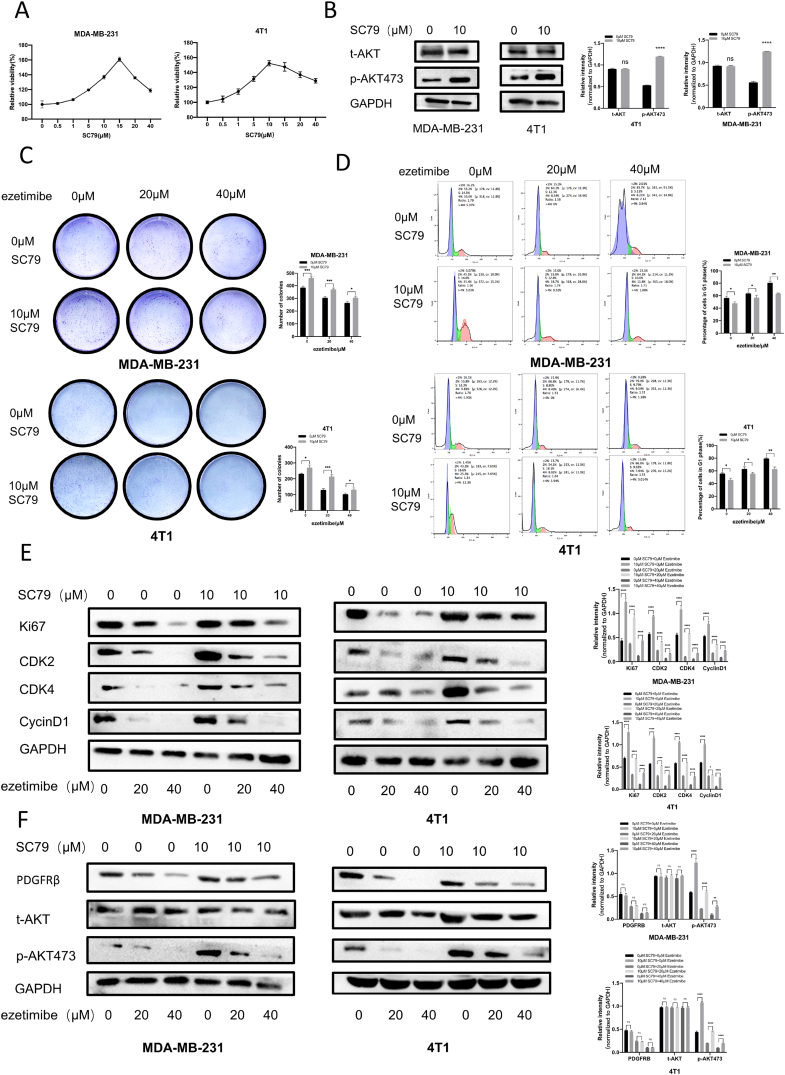
**The effects of AKT activation on the inhibition of MDA-MB-231 and 4T1 cell proliferation and cell cycle arrest by Ezetimibe.** (A) MDA-MB-231 and 4T1 cells were treated with different concentrations of SC79 (0, 0.5, 1, 5, 10, 15, 20, 40 μmol/L) for 48 h, and cell viability was measured using the CCK-8 assay. (B) MDA-MB-231 and 4T1 cells were treated with SC79 (0, 10 μmol/L) for 48 h, and the levels of *t*-AKT and *p*-AKT473 were detected using Western blotting analysis. (C) MDA-MB-231 cells were treated with different concentrations of Ezetimibe (0, 20, 40 μmol/L) and SC79 (0, 10 μmol/L) for 10 days, and 4T1 cells were treated for 14 days. The cell colony formation assay was used to measure the number of cell colonies formed. (D) MDA-MB-231 and 4T1 cells were treated with different concentrations of Ezetimibe (0, 20, 40 μmol/L) and SC79 (0, 10 μmol/L) for 48 h, and cell cycle distribution was analyzed using flow cytometry. (E) MDA-MB-231 and 4T1 cells were treated with different concentrations of Ezetimibe (0, 20, 40 μmol/L) and SC79 (0, 10 μmol/L) for 48 h, and the expression levels of Ki67, CDK2, CDK4, and CyclinD1 proteins were determined by Western blotting analysis. (F) MDA-MB-231 and 4T1 cells were treated with different concentrations of Ezetimibe (0, 20, 40 μmol/L) and SC79 (0, 10 μmol/L) for 48 h, and the levels of PDGFRβ, *t*-AKT, and *p*-AKT473 proteins were measured by Western blotting analysis. The results shown in the figure are representative of three independent experiments and are presented as mean ± standard deviation (Mean ± SD): *p < 0.05, **p < 0.01, ***p < 0.001, ****p < 0.0001. Here 0 μmol/L Ezetimibe, SC79 or other chemicals refers to 0.1 % (v/v) DMSO solution.

To explore the relationship between the inhibitory effects on TNBC cell proliferation and cell cycle arrest of TNBC cells by Ezetimibe and the AKT pathway, we utilized the AKT-specific activator SC79. CCK8 experiments demonstrated that 10 μM SC79 promoted the growth of MDA-MB-231 and 4T1 cells. Western blotting experiments showed that treatment with 10 μM SC79 successfully activated AKT in MDA-MB-231 and 4T1 cells, leading to a significant increase in phosphorylated AKT levels (Fig. 4A and B). Therefore, in this study, we selected 10 μM SC79 to activate AKT in MDA-MB-231 and 4T1 cells. The results of the cell clone formation experiment indicated that the number of clones formed by SC79-stimulated MDA-MB-231 and 4T1 TNBC cells treated with different concentrations of Ezetimibe was significantly higher than that of the control cells treated with same concentrations of Ezetimibe (Fig. 4C). This suggests that the activation of AKT in TNBC cells significantly counteracts the inhibitory effect of Ezetimibe on cell proliferation. Furthermore, the number of cells arrested in the G1 phase after treatment with different concentrations of Ezetimibe was significantly reduced in SC79-stimulated MDA-MB-231 and 4T1 TNBC cells compared to the control cells treated with same concentrations of Ezetimibe (Fig. 4D). This indicates that the activation of AKT in TNBC cells significantly weakens the ability of Ezetimibe to arrest cells in the G1/S phase. Compared to the control cells treated with different concentrations of Ezetimibe, the levels of Ki67, CDK2, CDK4, and CyclinD1 proteins significantly increased in SC79-stimulated MDA-MB-231 and 4T1 TNBC cells treated with same concentrations of Ezetimibe (Fig. 4E). These results confirm that the activation of AKT in TNBC cells significantly reverses the inhibitory effects of Ezetimibe on proliferation and cell cycle-related proteins. It also verifies that the activation of AKT in TNBC cells significantly weakens the inhibitory effect of Ezetimibe on cell proliferation and the ability to arrest cells in the G1/S phase. To investigate how AKT activation in TNBC cells affects the action of Ezetimibe on the PDGFRβ/AKT pathway, the expression of PDGFRβ/AKT pathway-related proteins in the cells was examined through Western blotting experiments. The protein expression level of *p*-AKT473 in TNBC cells treated simultaneously with SC79 and Ezetimibe significantly increased compared to the control group treated with Ezetimibe alone, while there was no significant difference in the expression level of PDGFRβ protein between the two groups of cells, and its expression was not affected by AKT activation (Fig. 4F).

These results demonstrate that AKT activation can reverse the inhibitory effect of Ezetimibe on the activation of the AKT pathway in TNBC cells. It also confirms that PDGFRβ protein serves as an upstream protein of AKT, indicating that AKT is a key target for Ezetimibe in inhibiting TNBC cell proliferation.

The AKT inhibitor MK2206 enhances the inhibitory effect of Ezetimibe on the proliferation and cell cycle arrest of PDGFRβ-overexpressing TNBC cells.

**Fig. 5:**
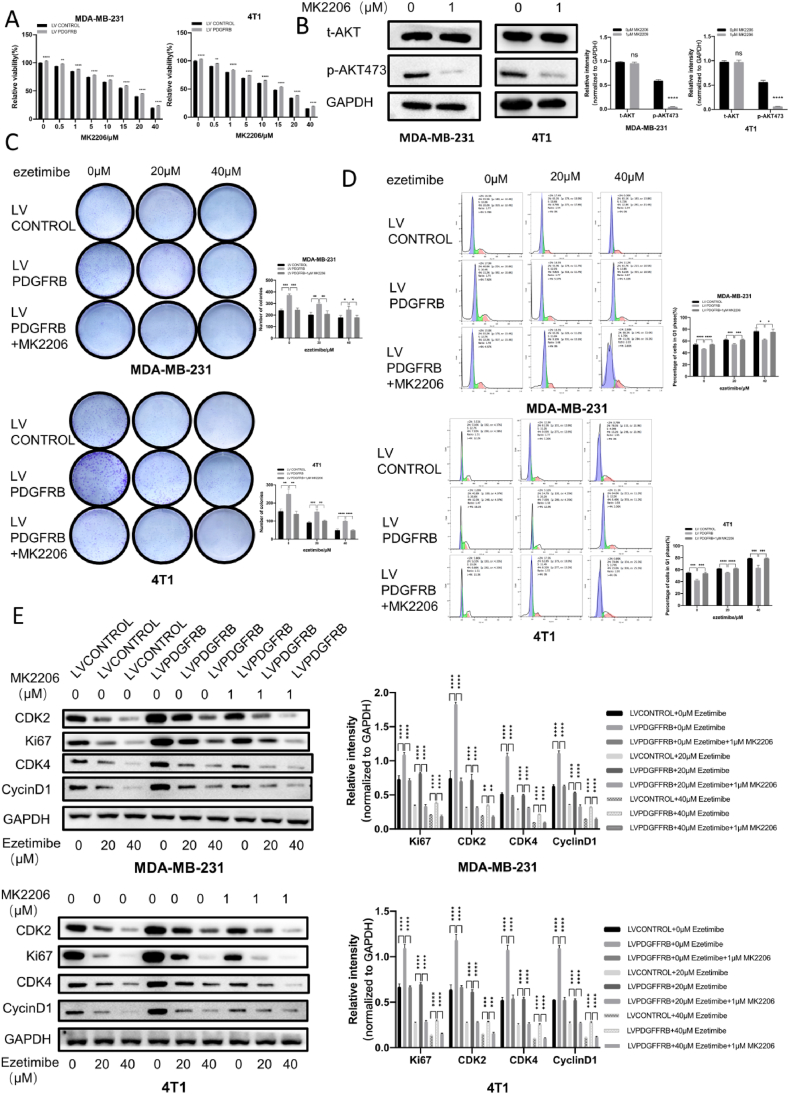

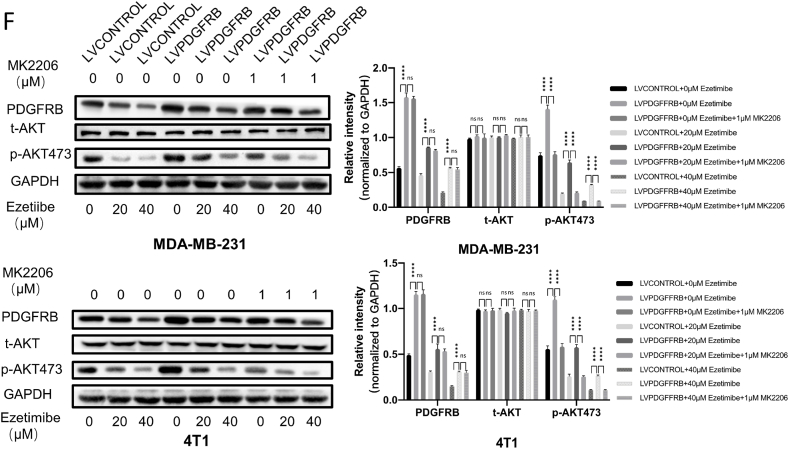
**The impact of MK2206 on the inhibitory effect on the proliferation and cell cycle arrest of PDGFRβ overexpressing TNBC cells by Ezetimibe.** (A) MDA-MB-231 and 4T1 cells overexpressing PDGFRβ or transfected with empty vector were treated with different concentrations of MK2206 (0, 0.5, 1, 5, 10, 15, 20, 40 μmol/L) for 48 h, and cell viability was measured using the CCK-8 assay. (B) MDA-MB-231 and 4T1 cells were treated with MK2206 (0, 1 μmol/L) for 48 h, and the expression of *t*-AKT and *p*-AKT473 was examined by Western blot. (C) MDA-MB-231 cells overexpressing PDGFRβ or transfected with empty vector were treated with different concentrations of MK2206 (0, 1 μmol/L) and Ezetimibe (0, 20, 40 μmol/L) for 10 days, and 4T1 cells were treated for 14 days. Cell clony formation was assessed by colony formation assay. (D) PDGFRβ-overexpressing MDA-MB-231 and 4T1 cells, as well as control cells, were treated with different concentrations of MK2206 (0, 1 μmol/L) and Ezetimibe (0, 20, 40 μmol/L) for 48 h, and cell cycle distribution was analyzed by flow cytometry. (E) PDGFRβ-overexpressing MDA-MB-231 and 4T1 cells, as well as control cells, were treated with different concentrations of MK2206 (0, 1 μmol/L) and Ezetimibe (0, 20, 40 μmol/L) for 48 h, and the expression of Ki67, CDK2, CDK4, and CyclinD1 proteins was examined by Western blot. (F) PDGFRβ-overexpressing MDA-MB-231 and 4T1 cells, as well as control cells, were treated with different concentrations of MK2206 (0, 1 μmol/L) and Ezetimibe (0, 20, 40 μmol/L) for 48 h, and the expression of PDGFRβ, *t*-AKT, and *p*-AKT473 proteins was examined by Western blot. The results shown in the figures represent the mean ± standard deviation (Mean ± SD) of three independent experiments. Statistical significance is denoted as: *p < 0.05, **p < 0.01, ***p < 0.001, ****p < 0.0001. Here 0 μmol/L Ezetimibe, MK2206 or other chemicals refers to 0.1 % (v/v) DMSO solution.

In this study, we selected the AKT-specific inhibitor MK2206 to investigate whether targeted inhibition of AKT could restore the inhibitory effect on cell proliferation and cell cycle arrest by Ezetimibe in TNBC cells overexpressing PDGFRβ. First, we examined the effect of different concentrations of MK2206 on the viability of TNBC cells. As shown in Fig. 5A, 1 μM MK2206 exhibited weaker toxicity toward TNBC cells overexpressing PDGFRβ compared to control TNBC cells. Additionally, Western blotting experiments confirmed a significant decrease in phosphorylated AKT levels in MDA-MB-231 and 4T1 cells treated with 1 μM of MK2206 (Fig. 5B). Therefore, we selected 1 μM of MK2206 to treat MDA-MB-231 and 4T1 cells.

Cell clone formation assays demonstrated that TNBC cells overexpressing PDGFRβ treated with both Ezetimibe and MK2206 formed significantly fewer colonies than TNBC cells overexpressing PDGFRβ treated with Ezetimibe and DMSO solution simultaneously (Fig. 5C). These results indicate that inhibition of AKT activity can restore the ability of Ezetimibe to inhibit cell proliferation in TNBC cells overexpressing PDGFRβ. Flow cytometry analysis of the cell cycle showed that TNBC cells treated with both Ezetimibe and MK2206 exhibited a significantly higher number of cells arrested in the G1 phase than control cells treated with Ezetimibe and DMSO solution simultaneously (Fig. 5D). This suggests that inhibition of AKT activity can restore Ezetimibe's ability to arrest the cell cycle at the G1/S phase in TNBC cells overexpressing PDGFRβ. Compared to TNBC cells overexpressing PDGFRβ treated with Ezetimibe and DMSO solution simultaneously, those treated with both Ezetimibe and MK2206 showed a significant decrease in the levels of Ki67, CDK2, CDK4, and Cyclin D1 proteins (Fig. 5E).

These results validate that in TNBC cells overexpressing PDGFRβ, inhibition of AKT activity enhances the inhibitory effect on cell proliferation and cell cycle arrest by Ezetimibe. The level of *p*-AKT473 was significantly reduced in PDGFRβ overexpressing TNBC cells treated with both Ezetimibe and MK2206 compared to those treated with Ezetimibe and DMSO solution simultaneously. However, there was no significant difference in the expression level of PDGFRβ protein between these two groups, indicating that the expression of PDGFRβ protein is not affected by AKT inhibition (Fig. 5F).

These results further elucidate that the PDGFRβ/AKT signaling pathway is a crucial pathway for the inhibitory effect of Ezetimibe on TNBC proliferation and cell cycle arrest.

#### 3.2.1 Ezetimibe inhibits the growth of TNBC in vivo through the PDGFRβ/AKT pathway

**Fig. 6:**
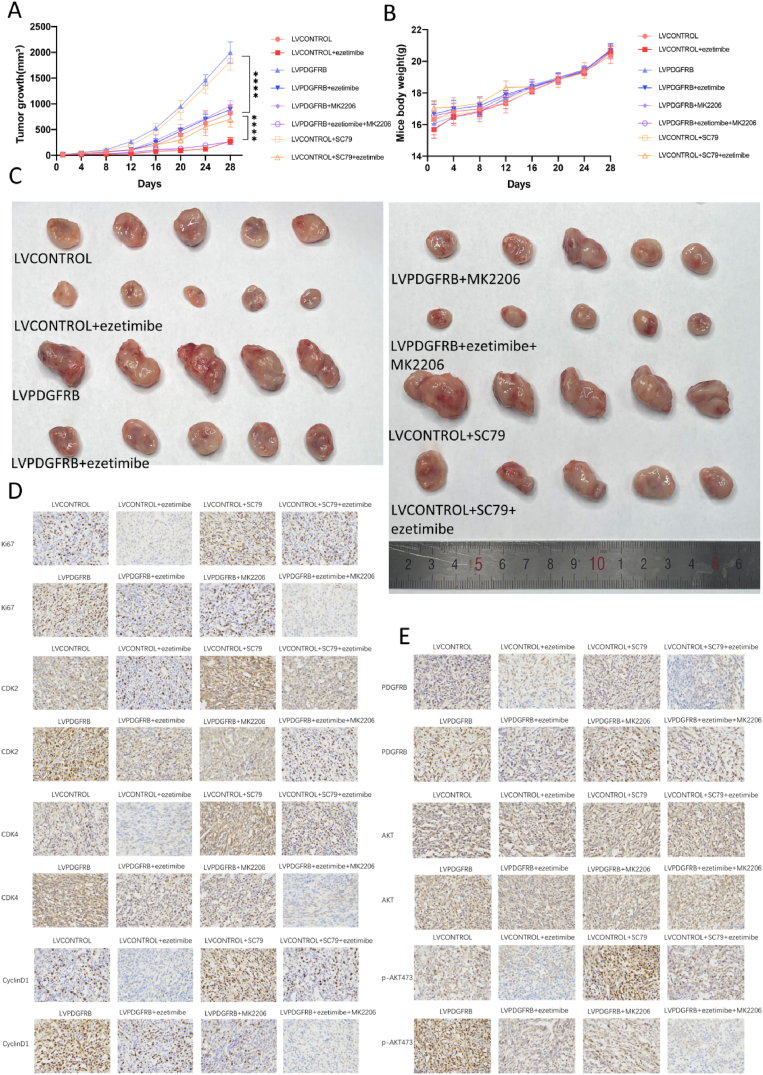
**The effects of Ezetimibe on TNBC growth and the PDGFRβ/AKT pathway in vivo.** (A) MDA-MB-231 cells overexpressing PDGFRβ or transfected with empty vector were separately used to establish subcutaneous breast tumor models in mice. When the tumors reached a size of 5 mm in length, the mice were randomly divided into 8 groups, with 5 mice in each group. The groups received the following treatments: LVCONTROL (empty vector MDA-MB-231) with vehicle treatment, LVCONTROL + ezetimibe (empty vector MDA-MB-231) treated with ezetimibe alone, LVPDGFRB (PDGFRβ-overexpressing MDA-MB-231) with vehicle treatment, LVPDGFRB + ezetimibe (PDGFRβ-overexpressing MDA-MB-231) treated with ezetimibe alone, LVPDGFRB + MK2206 (PDGFRβ-overexpressing MDA-MB-231) treated with MK2206 alone, LVPDGFRB + ezetimibe + MK2206 (PDGFRβ-overexpressing MDA-MB-231) treated with the combination of ezetimibe and MK2206, LVCONTROL + SC79 (empty vector MDA-MB-231) treated with SC79 alone, and LVCONTROL + ezetimibe + SC79 (empty vector MDA-MB-231) treated with the combination of ezetimibe and SC79. During the treatment process, the body weight (A) and tumor length and width (B) were measured at regular intervals. The tumor volume was calculated using the formula: length × width^2^/2. (C) When the tumor volume reached 2000 mm^3^, the mice were euthanized while under anesthesia, and the tumors were harvested and photographed. (D) Tumors from xenograft mice with breast cancer were harvested when they reached a size of 2000 mm^3^. Immunohistochemical staining was performed on the xenograft tumors from each group to detect the expression levels of Ki67, CDK2, CDK4, and CyclinD1. Representative images were captured under a microscope at a magnification of × 400. (E) Immunohistochemical staining was performed on the xenograft tumors from each group to detect the expression levels of PDGFRβ, AKT, and *p*-AKT473. Representative images were captured under a microscope at a magnification of × 400. The results presented in the figures are representative of three independent experiments and are expressed as mean ± standard deviation (Mean ± SD): *p < 0.05, **p < 0.01, ***p < 0.001, ****p < 0.0001.

In the nude mouse TNBC xenograft model, no animal deaths were observed in any of the treatment groups, and there were no significant differences in mouse weight (Fig. 6A). This indicates that 20 mg/kg Ezetimibe, 10 mg/kg SC79, and 10 mg/kg MK2206 have no significant drug toxicity in mice.

Among the four groups of subcutaneous tumor mice with empty vector cells, the tumor growth rate in the Ezetimibe treatment group was significantly slower than that in the vehicle control group (Fig. 6B). The tumor growth rate in the SC79 treatment group was significantly faster than that in the vehicle control group (Fig. 6B). The subcutaneous tumor growth rate in the Ezetimibe combined with SC79 treatment group was significantly lower than that in the SC79 treatment group alone but significantly higher than that in the Ezetimibe treatment group alone (Fig. 6B).

The tumor growth rate in mice with PDGFRβ-overexpressing TNBC cells was significantly faster than that in the control cell group (Fig. 6B).

Among the four groups of subcutaneous tumor mice constructed with PDGFRβ-overexpressing cells, the tumor growth rate in the Ezetimibe or MK2206 treatment group alone was significantly slower than that in the vehicle control group, and the tumor growth rate in the Ezetimibe combined with MK2206 treatment group was significantly slower than in the other three groups (Fig. 6B).

Fig. 6C demonstrated similar trends in tumor volume changes as observed in Fig. 6B.

Furthermore, the expression of the proliferation marker Ki67, and the cell cycle markers CDK2, CDK4, and CyclinD1 was analyzed through immunohistochemical detection of tumor samples. The results shown in Fig. 6D indicated that the changes in proliferation and cell cycle markers were consistent with the changes in tumor volume observed in Fig. 6A. These results suggest that Ezetimibe or MK2206 alone significantly inhibits the in vivo growth of TNBC cells. Overexpression of PDGFRβ and SC79 alone significantly promotes the in vivo growth of TNBC cells and this promotion can inhibit the anti-tumor proliferative effect of Ezetimibe in TNBC mice, whereas the addition of MK2206 can counteract the inhibitory effect of PDGFRβ overexpression on the therapeutic efficacy of Ezetimibe.

The expression of PDGFRβ, AKT, and *p*-AKT473, proteins related to the PDGFRβ/AKT pathway, was examined through immunohistochemical detection of tumors.

In the four groups of subcutaneous tumor models constructed with empty vector cells, the expression levels of PDGFRβ and *p*-AKT473 in the Ezetimibe treatment group alone were significantly lower than those in the vehicle control group and the SC79 treatment group alone. The expression levels of PDGFRβ and *p*-AKT473 in the Ezetimibe combined with SC79 treatment group were significantly lower than those in the SC79 treatment group alone, but the expression level of *p*-AKT473 in the Ezetimibe combined with SC79 treatment group was significantly higher than that in the Ezetimibe treatment group alone. The expression levels of PDGFRβ in the SC79 treatment group alone and the vehicle control group were significantly lower than those in the PDGFRβ-overexpressing group treated with vehicle. The expression levels of *p*-AKT473 in the SC79 treatment group alone and the PDGFRβ-overexpressing group treated with the drug carrier were significantly higher than those in the control cell group treated with the vehicle.

In the four groups of subcutaneous tumor models constructed with PDGFRβ-overexpressing cells, the expression of PDGFRβ in the Ezetimibe treatment group alone and the Ezetimibe combined with MK2206 treatment group was significantly lower than that in the MK2206 treatment group alone or the vehicle control group. The levels of *p*-AKT473 in the Ezetimibe treatment group alone and the MK2206 treatment group alone were significantly lower than those in the vehicle control group, while the expression levels of *p*-AKT473 in the Ezetimibe combined with MK2206 treatment group were significantly lower than those in the other three groups (Fig. 6E).

In summary, Ezetimibe exhibits a significant in vivo anti-tumor effect and exerts this effect by inhibiting the PDGFRβ/AKT pathway.

## 4 Discussion

In recent years, there has been some progress in the study of drugs that target cholesterol metabolism in breast cancer growth [18]. Khandan Keyomarsi et al. found that lovastatin, a statin that inhibits cholesterol synthesis, inhibits the growth of breast cancer cells and causes cells to develop G1 phase arrest, during which the expression of CylinD1 protein is reduced [35]. Stacy W Blain found that a decrease in the CylinD1 protein led to the release of p27 from the CDK4/6-CylinD1 complex, while it assembled with CDK2 to inhibit CDK2, thereby blocking the cell cycle to inhibit proliferation [36]. Our study found that ezetimibe can also inhibit the growth of breast cancer cells and block cells in the G1 phase, while inhibiting the expression of CDK2, CDK4 and CyclinD1 proteins, so we speculate that ezetimibe-induced G1 blockade of breast cancer cells may occur by inhibiting CyclinD1 and releasing p27 in CDK4/6-CylinD1 complex, inducing the upregulation of p27 to inhibit CDK2, thereby inhibiting the proliferation of breast cancer cells.

Mahiro Iizuka-Ohashi et al. found that fluvastatin and simvastatin can promote apoptosis of breast cancer cells and inhibit cell proliferation, thereby inhibiting breast cancer growth, but this inhibition depends on the inhibition of the downstream isoprene-gerani-geraniyl pyrophosphate (GGPP), which in turn inhibits the activation of AKT [37]. This suggests that the inhibition of breast cancer cell proliferation by ezetimibe may be related not only to the pathway that suppresses cholesterol uptake, but also to the mechanism of breast cancer growth. We performed RNA-seq sequencing of ezetimibe-treated breast cancer cells, and the results showed that ezetimibe inhibited the expression of PDGFRβ in TNBC cells. Existing drug studies targeting PDGFRβ have shown that reducing the expression of PDGFRβ in cells can inhibit tumor progression. Imatinib is an inhibitor targeting PDGFRβ tyrosine kinase receptors, Marion T Weigel et al. found that imatinib inhibits the growth of breast cancer cells and induces apoptosis, while imatinib exerts its role in inhibiting growth by inhibiting the activity of PDGFRβ and AKT [38], and imatinib can improve the antitumor effect of chemotherapy drugs such as vinorelbine and paclitaxel [39]. Transcriptome sequencing analysis and in vitro studies showed that ezetimibe could significantly inhibit the expression of PDGFRβ and AKT in triple-negative breast cancer cells, suggesting that ezetimibe may enhance the therapeutic effect of antitumor drugs by inhibiting the PDGFRβ/AKT pathway. We also found that ezetimibe significantly inhibited AKT activation, which in TNBC cells is resistant to ezetimibe's inhibitory effects on tumor cell proliferation and cell cycle. Mahiro Iizuka-Ohashi et al. found that the lipophilic statins simvastatin and fluvastatin can inhibit the activation of AKT in breast cancer cells, thereby inducing apoptosis to inhibit tumor progression [37]. This suggests that ezetimibe suppresses breast cancer cell proliferation not only by inducing cell G1 phase arrest, but also by inducing apoptosis, thereby inhibiting breast cancer growth.

This study reveals for the first time a new mechanism by which ezetimibe acts on TNBC, that is, ezetimibe inhibits the growth of TNBC by inhibiting the activation of the PDGFRβ/AKT pathway, thereby inducing G1 blockade in tumor cells. Our study demonstrates the new use of the FDA-approved lipid-lowering drug ezetimibe for the treatment of tumors and provides a new theoretical basis for the application of drugs that regulate cholesterol metabolism to the treatment of tumors. Although we demonstrated that ezetimibe did not show growth inhibition and cycle arrest in pancreatic cancer (Supplementary Figs. 1A–C), more evidence is needed on whether ezetimibe is specific to TNBC, and whether there is an association with lipid metabolism. To further investigate this question, first, we need to screen out ezetimibe-sensitive and non-sensitive cell lines. Secondly, control cells and ezetimibe-treated cells undergo metabolomic sequencing [1] to compare differences in lipid metabolism between the two cell types [2] and to study the effect of ezetimibe on specific lipid metabolic pathways. It is worth noting that this article has a minor limitation. It has not been experimental demonstrated that a correlation exists between cholesterol levels and the progression of breast cancer. To address this matter, an investigation can be conducted to examine alterations in cholesterol levels during various stages of breast cancer progression, with the aim of elucidating any potential correlation. To assess the impact of low/high concentrations of cholesterol on breast cancer progression.

## Animal experiments and ethics

Animal experiments were approved by the Animal Ethical and Welfare Committee of NJU（SYXK2019-0056）

## Funding

The research was supported by the Shandong Provincial Laboratory Project (SYS202202); the 10.13039/501100001809National Natural Science Foundation of China (81972888, 82272819); the Research Project of Jinan Microecological Biomedicine Shandong Laboratory (JNL-202219 B, JNL-202204 A, JNL-2023017D); the Primary Research & Development Plan of 10.13039/501100002949Jiangsu Province (BE2022840); the Open Project of Chinese Materia Medica First-Class Discipline of 10.13039/501100007956Nanjing University of Chinese Medicine (2020YLXK007).

## Data availability statement

Data included in article/supp. Material/referenced in article; Data will be made available on request.

## CRediT authorship contribution statement

**Qinyu He:** Investigation. **Lingkai Kong:** Investigation. **Weiwei Shi:** Investigation. **Ding Ma:** Investigation. **Kua Liu:** Writing – original draft. **Shuwei Yang:** Investigation. **Qilei Xin:** Investigation. **Chunping Jiang:** Writing – review & editing, Conceptualization. **Junhua Wu:** Writing – review & editing, Conceptualization.

## Declaration of competing interest

The authors declare that they have no known competing financial interests or personal relationships that could have appeared to influence the work reported in this paper.
