# Supplementary material for: Ezetimibe inhibits triple-negative breast cancer proliferation and promotes cell cycle arrest by targeting the PDGFR/AKT pathway
Source: Heliyon. 2023 Oct 29;9(11):e21343. doi: 10.1016/j.heliyon.2023.e21343 (PMC10651468; doi:10.1016/j.heliyon.2023.e21343)
Supplement: Multimedia component 2 [file mmc2.pdf]

Fig1D

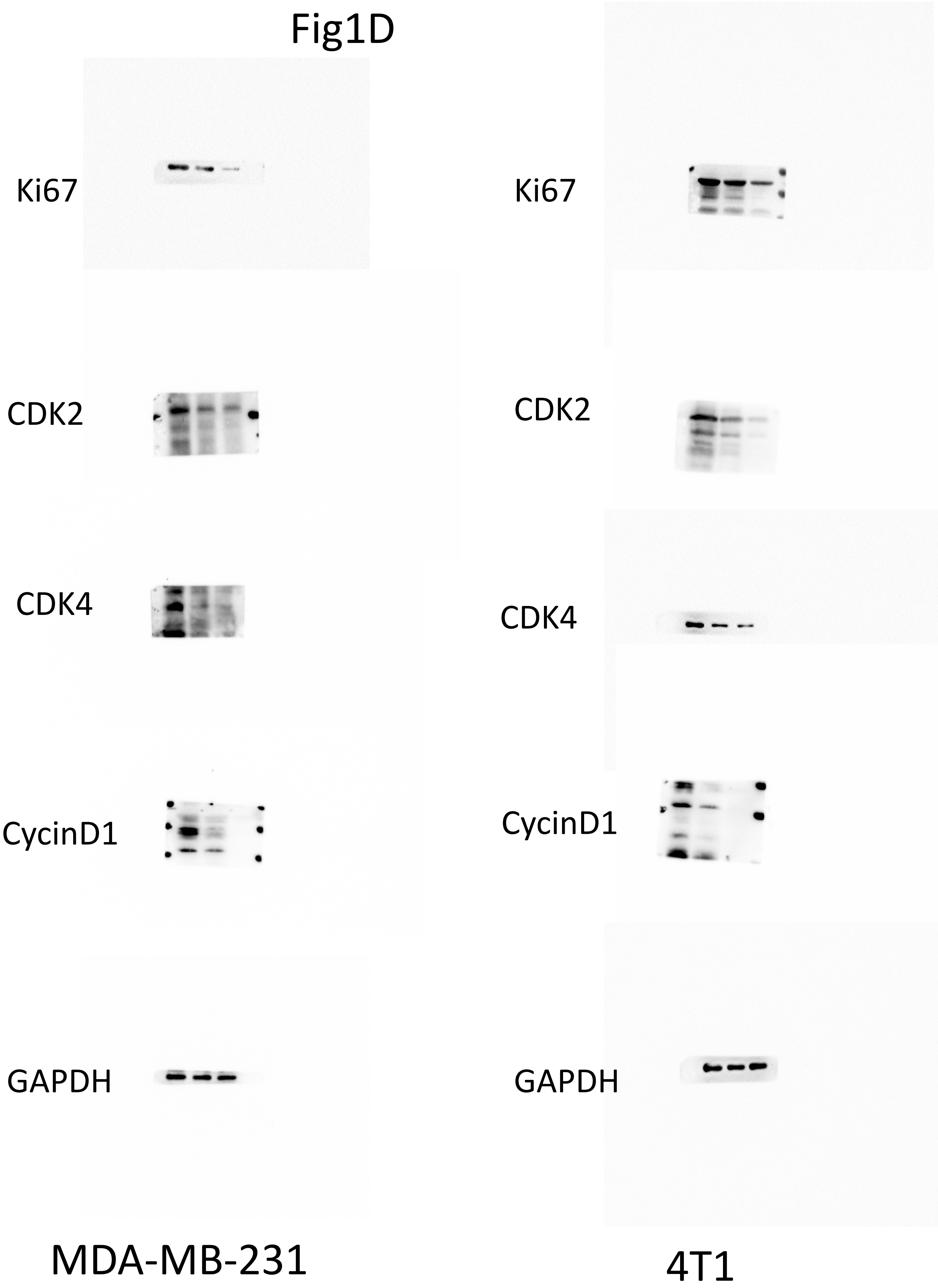
Ki67
Ki67
CDK2
CDK2
CDK4
CDK4
CycinD1
CycinD1
GAPDH
GAPDH
MDA-MB-231
4T1

Fig2F

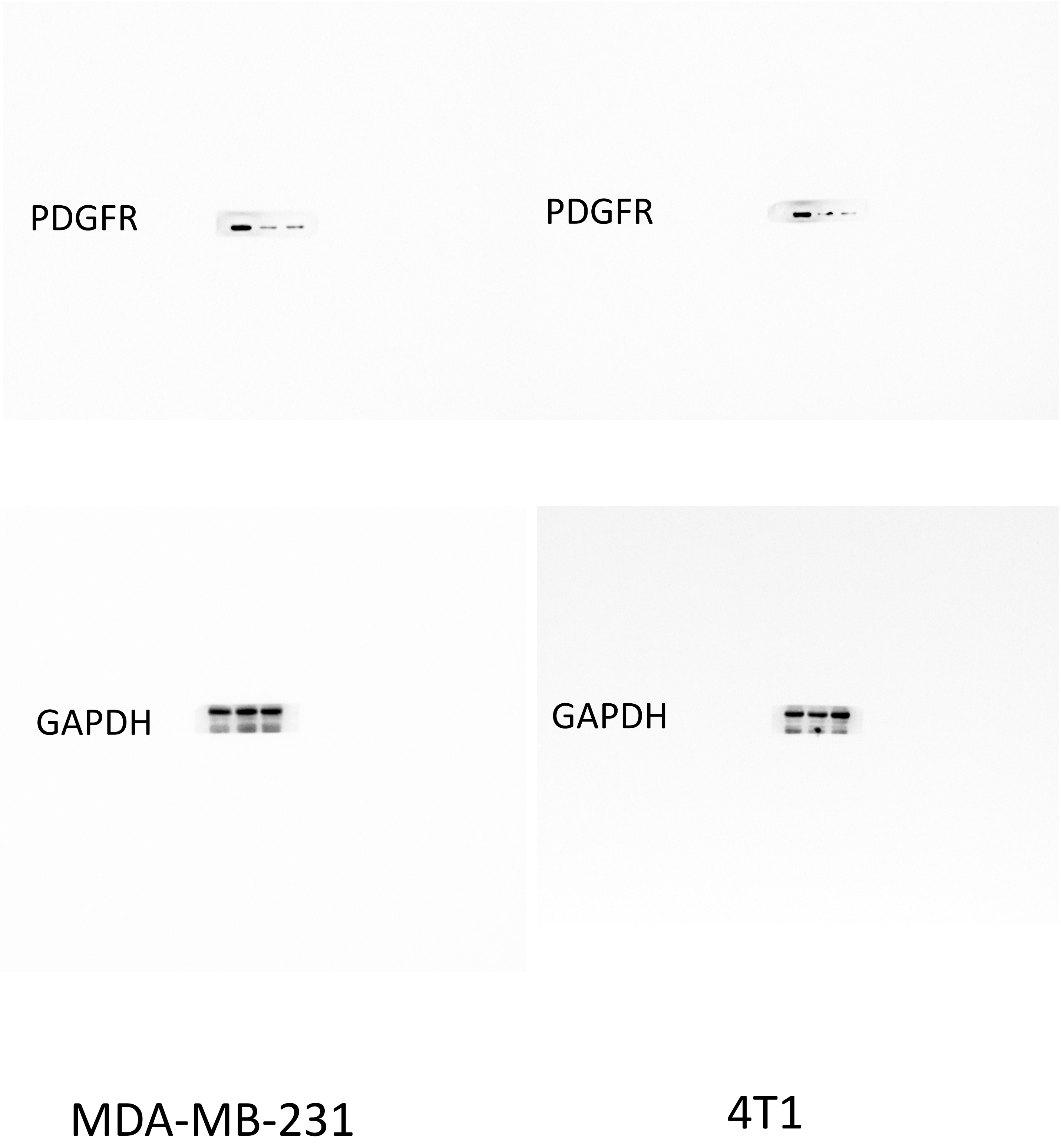

Fig3B

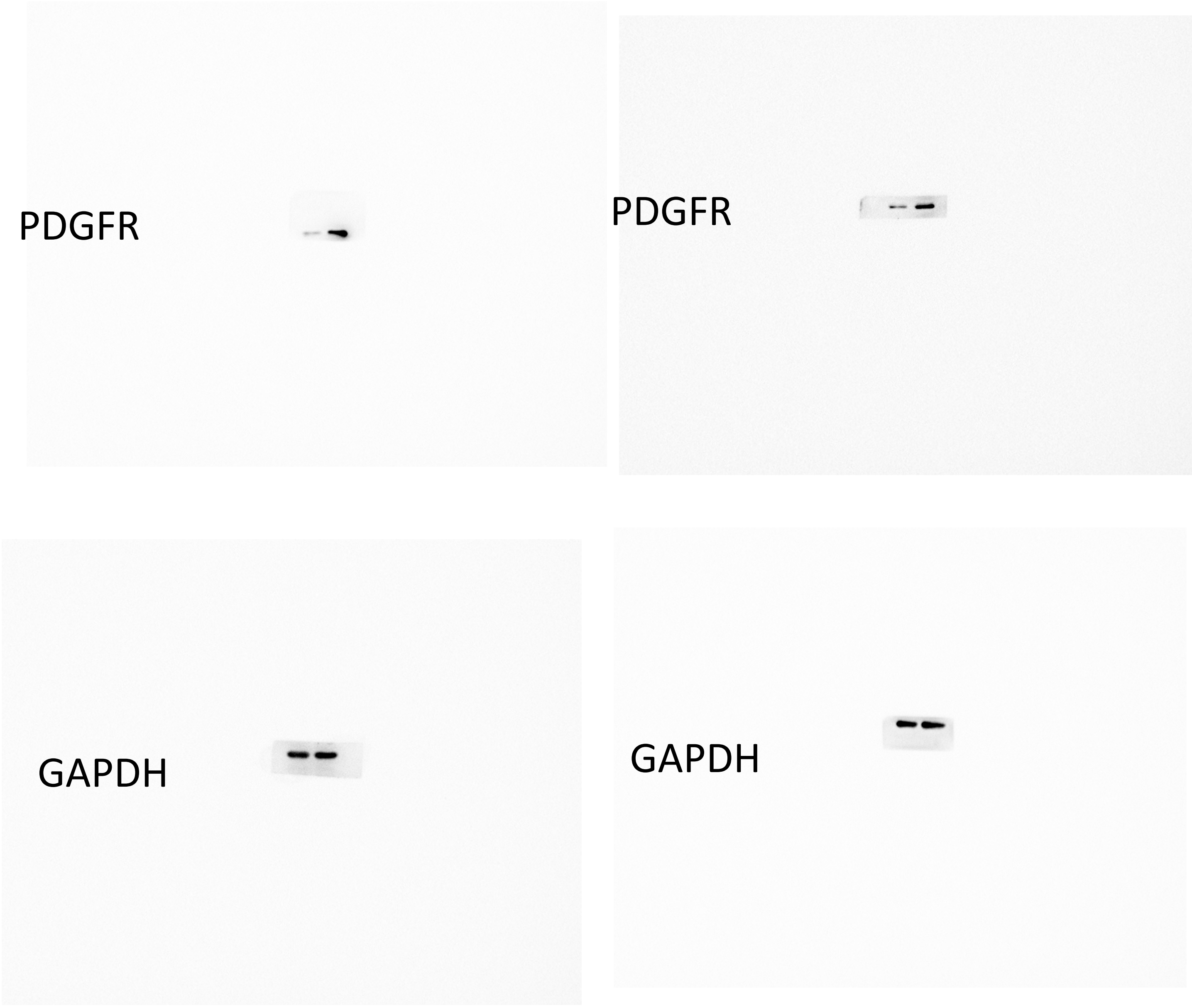

Fig3E

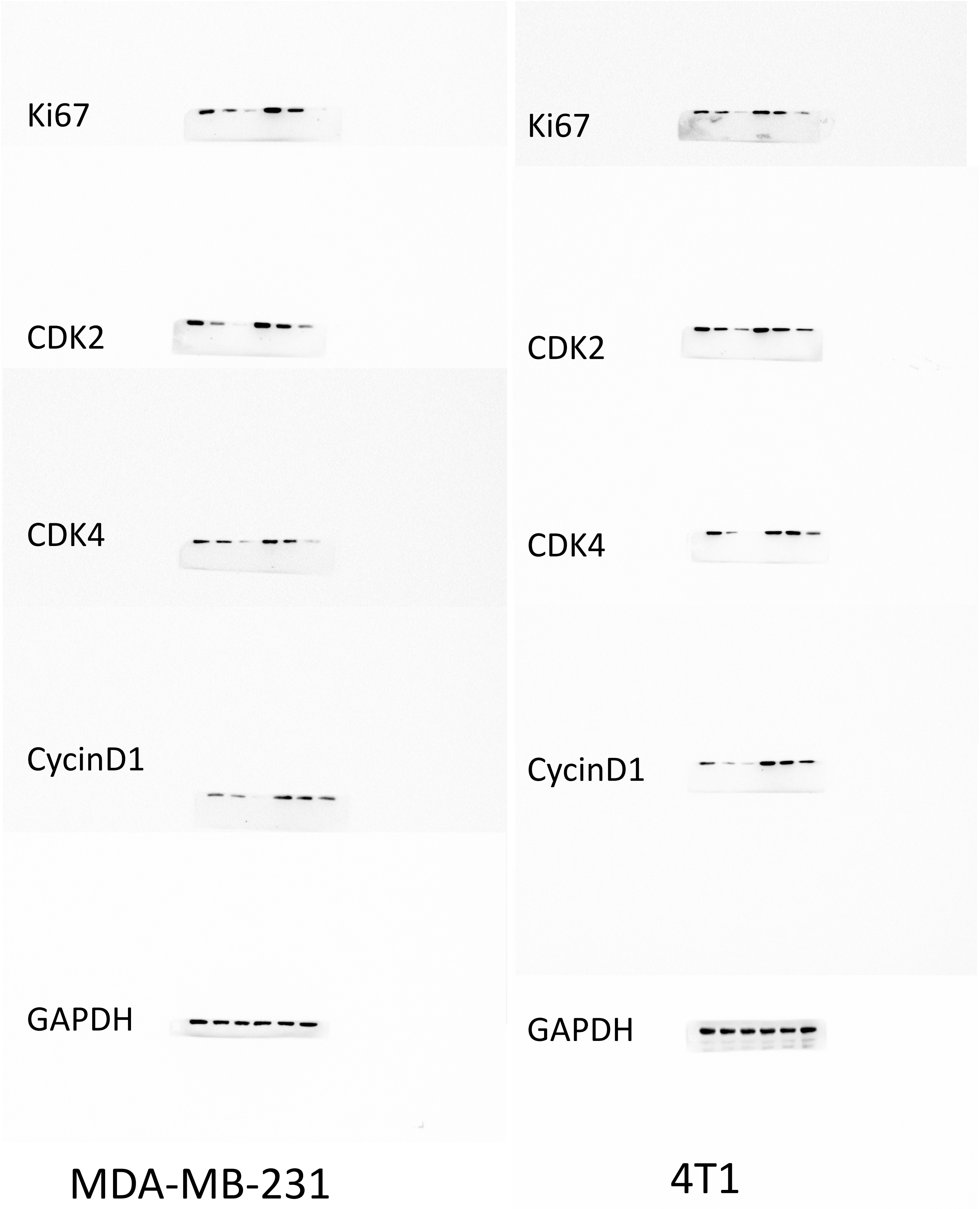
Ki67
CDK2
CDK4
CycinD1
GAPDH
MDA-MB-231
Ki67
CDK2
CDK4
CycinD1
GAPDH
4T1

Fig3F

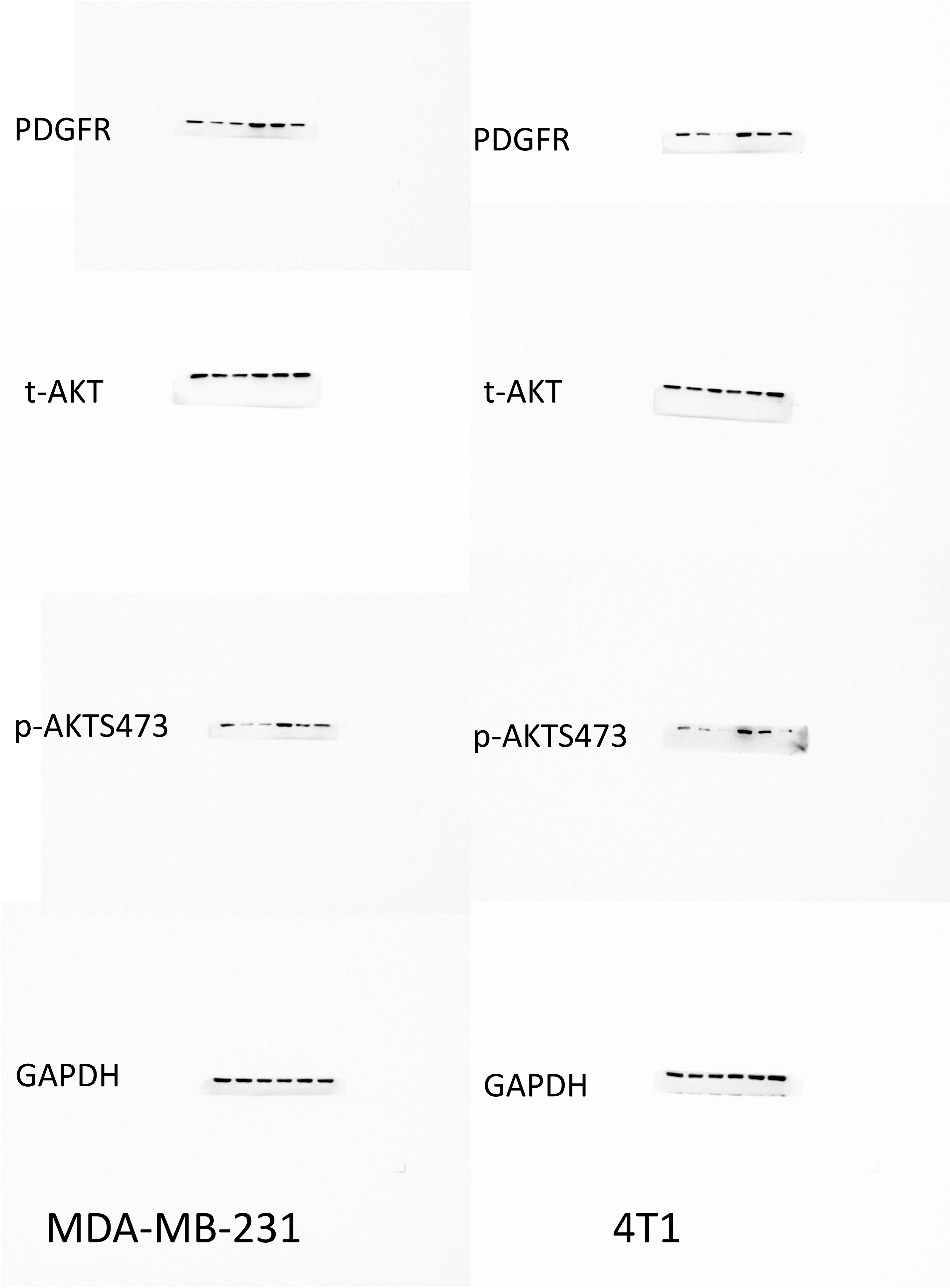
PDGFR
t-AKT
p-AKTS473
GAPDH
MDA-MB-231
PDGFR
t-AKT
p-AKTS473
GAPDH
4T1

Fig4B

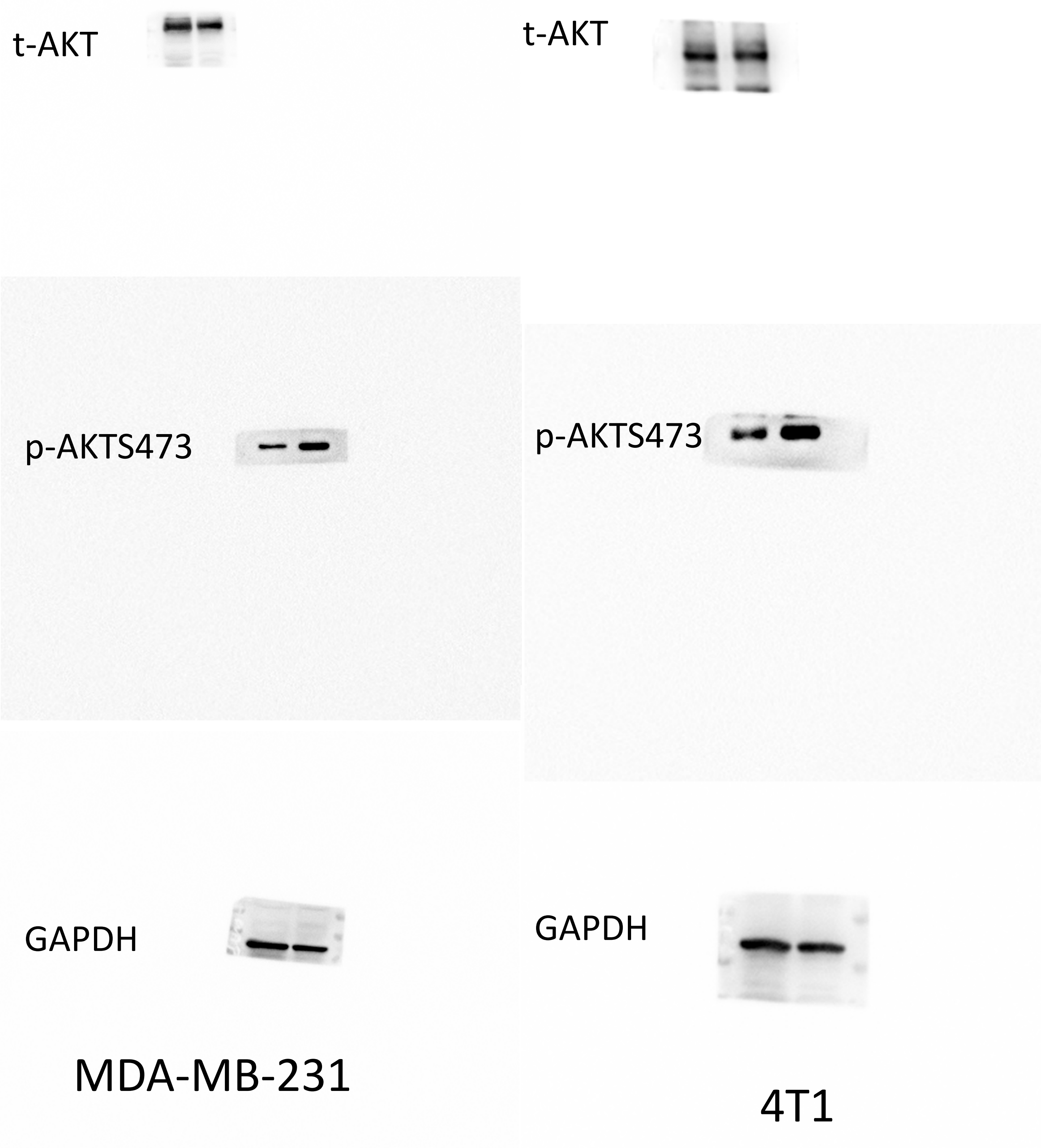

Fig4E

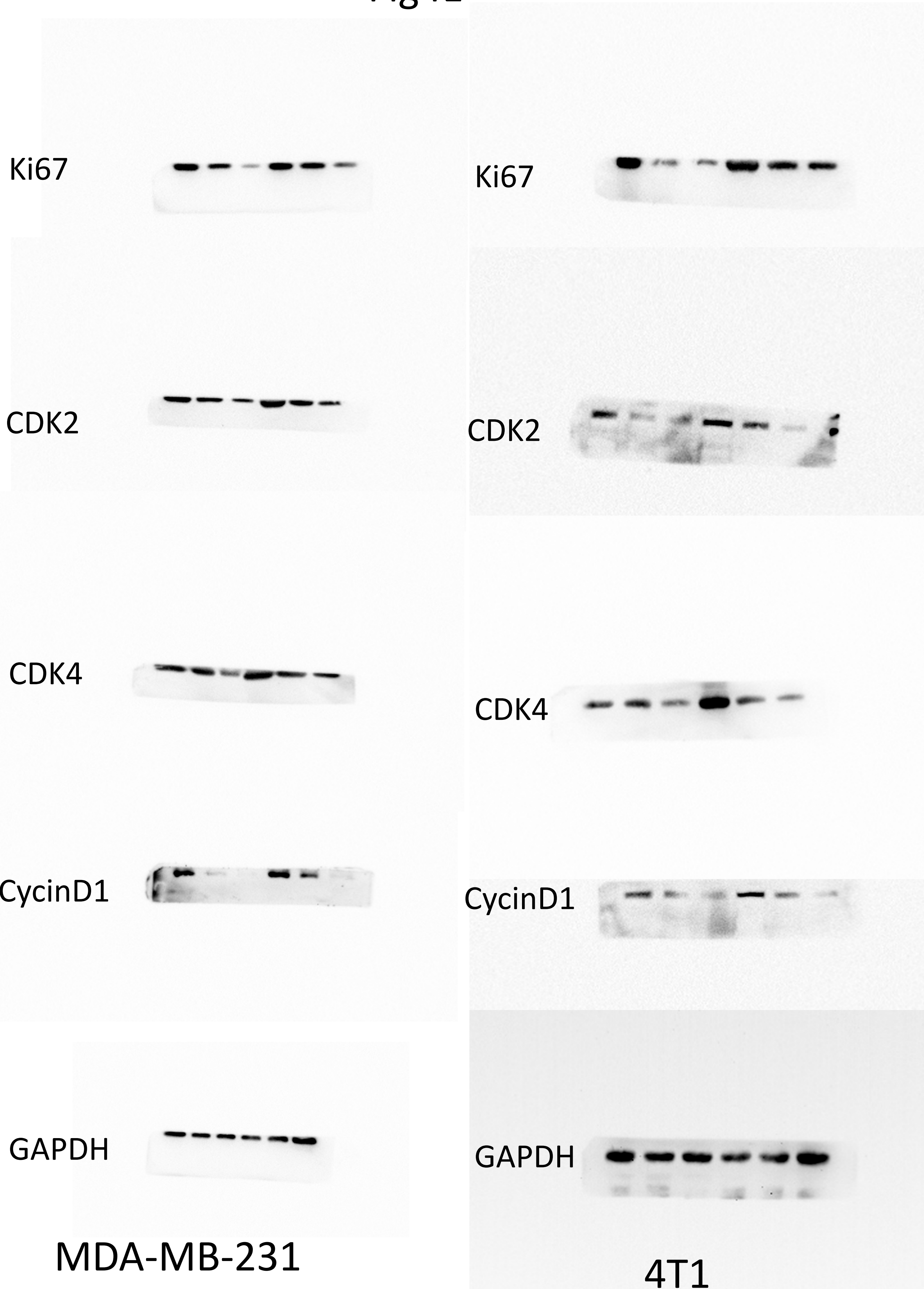
Ki67
CDK2
CDK4
CycinD1
GAPDH
MDA-MB-231
Ki67
CDK2
CDK4
CycinD1
GAPDH
4T1

Fig4F

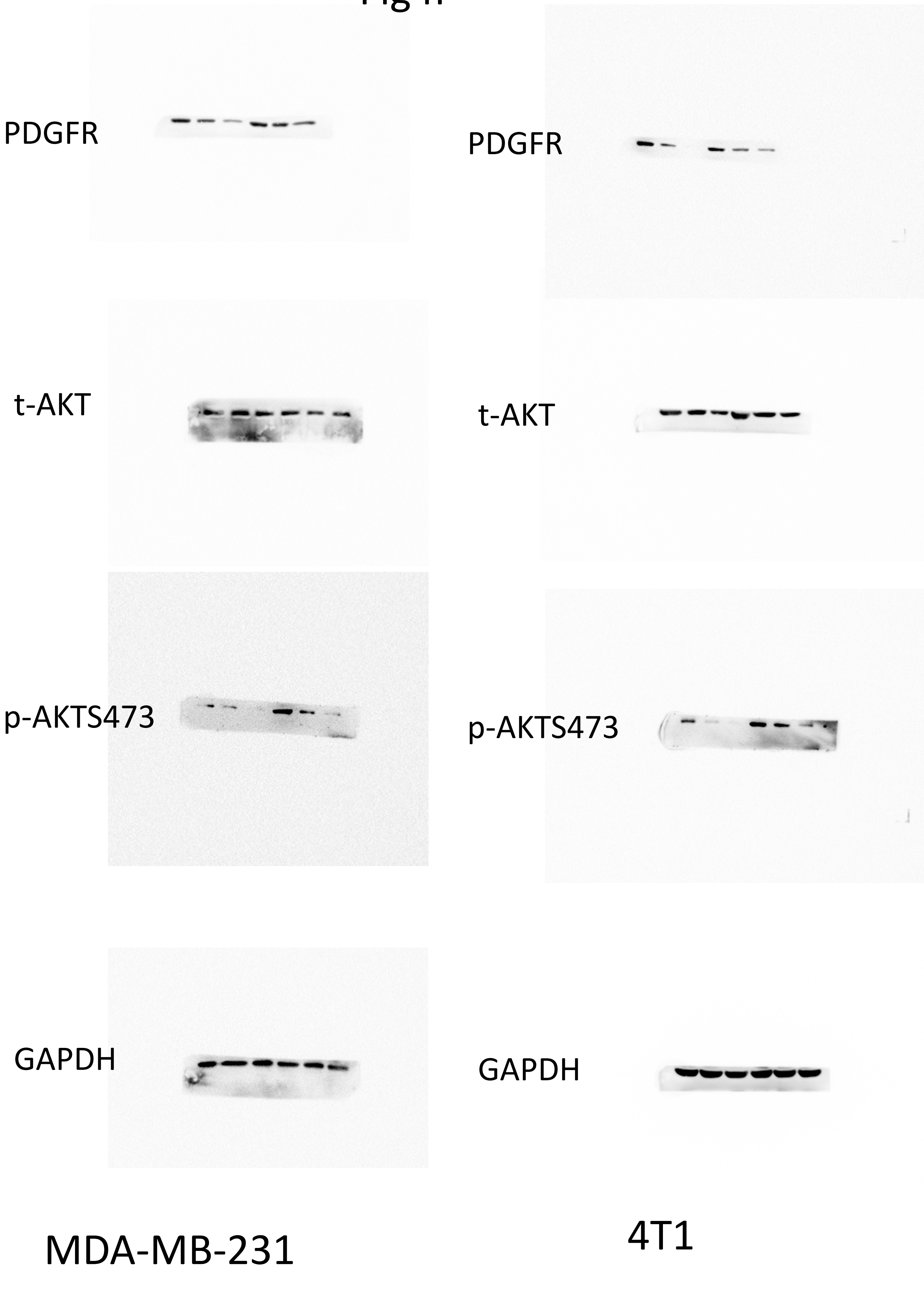
PDGFR
PDGFR
t-AKT
t-AKT
p-AKTS473
p-AKTS473
GAPDH
GAPDH
MDA-MB-231
4T1

Fig5B

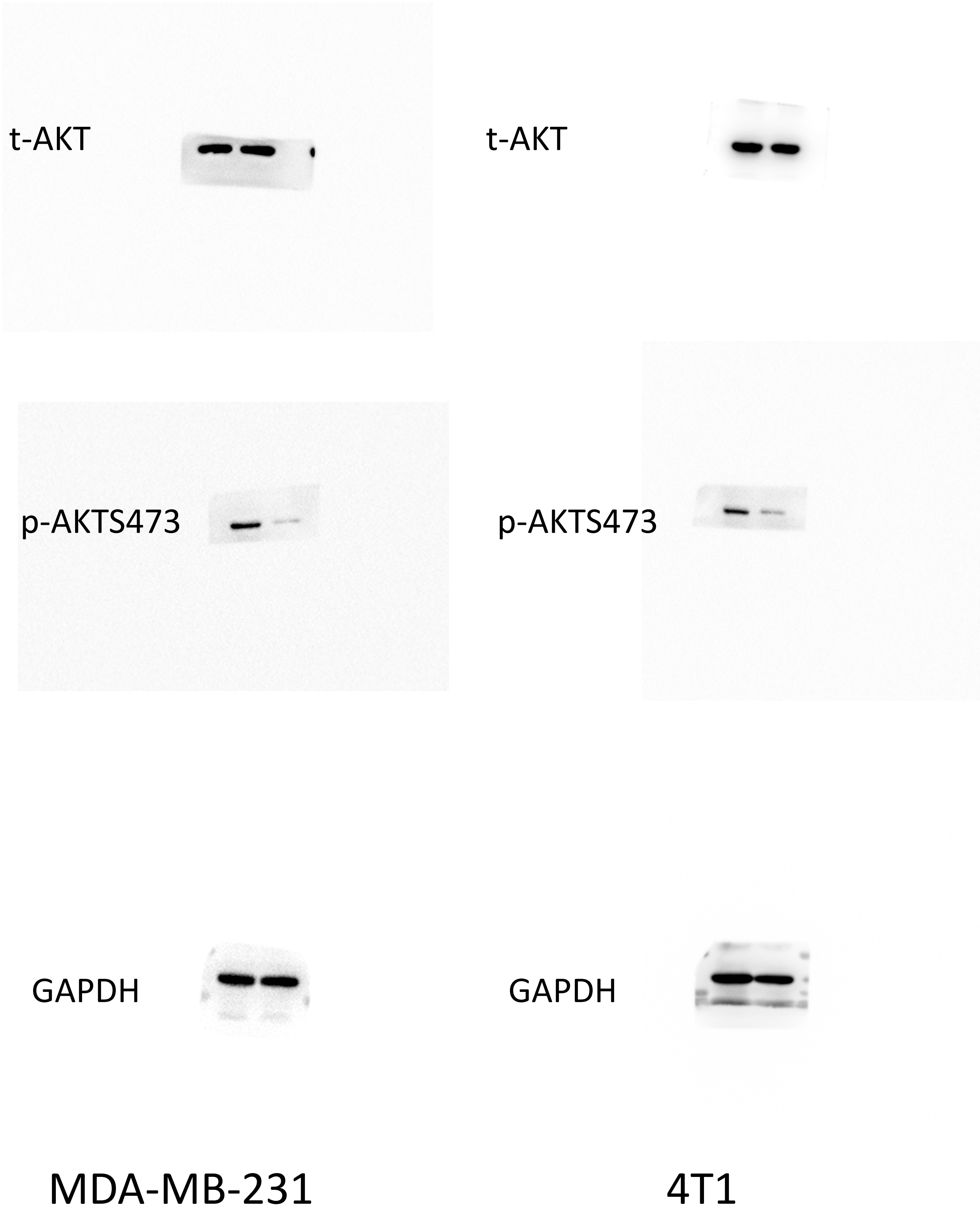

Fig5E

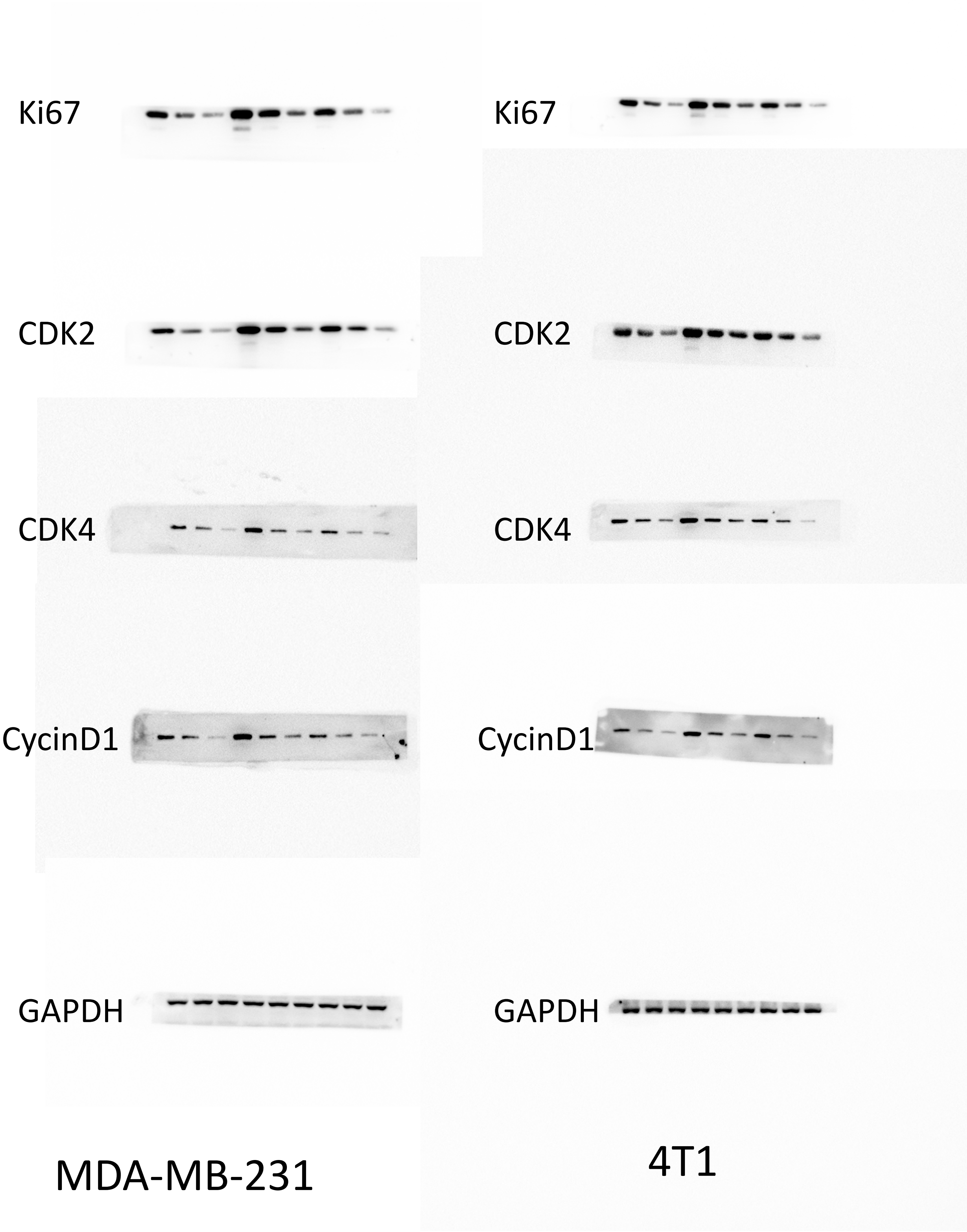
Ki67
Ki67
CDK2
CDK2
CDK4
CDK4
CycinD1
CycinD1
GAPDH
GAPDH
MDA-MB-231
4T1

Fig5F

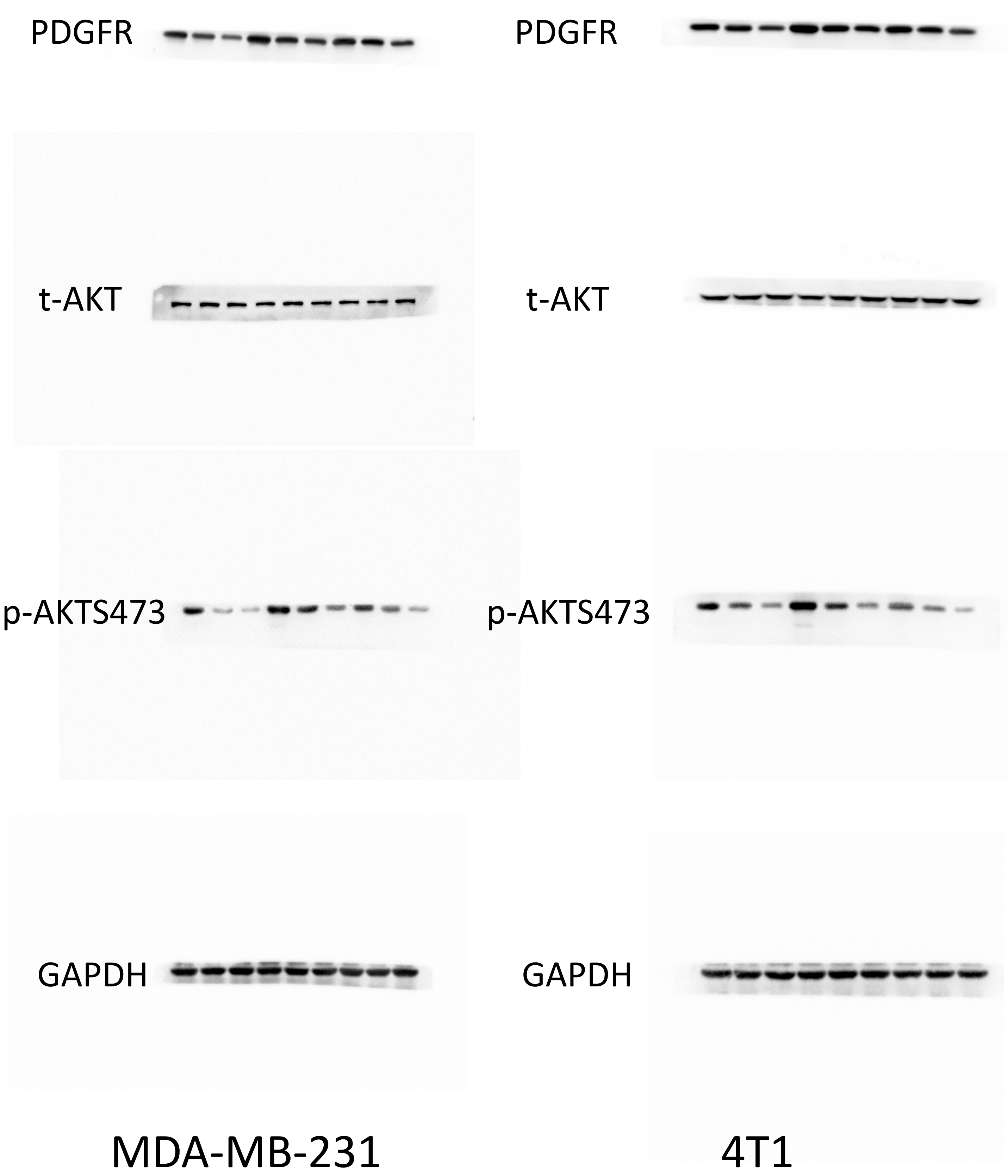
PDGFR
PDGFR
t-AKT
t-AKT
p-AKTS473
p-AKTS473
GAPDH
GAPDH
MDA-MB-231
4T1
