# Supplementary material for: Ezetimibe inhibits triple-negative breast cancer proliferation and promotes cell cycle arrest by targeting the PDGFR/AKT pathway
Source: Heliyon. 2023 Oct 29;9(11):e21343. doi: 10.1016/j.heliyon.2023.e21343 (PMC10651468; doi:10.1016/j.heliyon.2023.e21343)
Supplement: Multimedia component 3 [file mmc3.doc]

Supplementary Figure

**Ezetimibe inhibits** **Triple-Negative Breast Cancer proliferation and promotes cell cycle arrest by targeting the PDGFR/AKT pathway**

Qinyu He^2#^; Lingkai Kong^2#^; Weiwei Shi^2#^; Ding Ma^2, 3^; Kua Liu^2^; Shuwei Yang^2^; Qilei Xin^1^; Chunping Jiang^1, 2^*; Junhua Wu^1, 2^*

^1^Jinan Microecological Biomedicine Shandong Laboratory, Shounuo City Light West Block, Qingdao Road 3716#, Huaiyin District, Jinan City, Shandong Province, China.

^2^State Key Laboratory of Pharmaceutical Biotechnology, National Institute of Healthcare Data Science at Nanjing University, Jiangsu Key Laboratory of Molecular Medicine, the Affiliated Drum Tower Hospital of Nanjing University Medical School, Medical School, Nanjing University, 22 Hankou Road, Nanjing, Jiangsu, 210093 China.

^3^Department of Gastroenterology, Third Xiangya Hospital, Central South University, Changsha, Hunan, China.

^#^These authors contributed equally: Qinyu He, Lingkai Kong, Weiwei Shi. *Corresponding author: Junhua Wu **(**[wujunhua@nju.edu.cn](mailto:wujunhua@nju.edu.cn)**)** and Chunping Jiang **(**[chunpingjiang@nju.edu.cn](mailto:chunpingjiang@nju.edu.cn)**).**


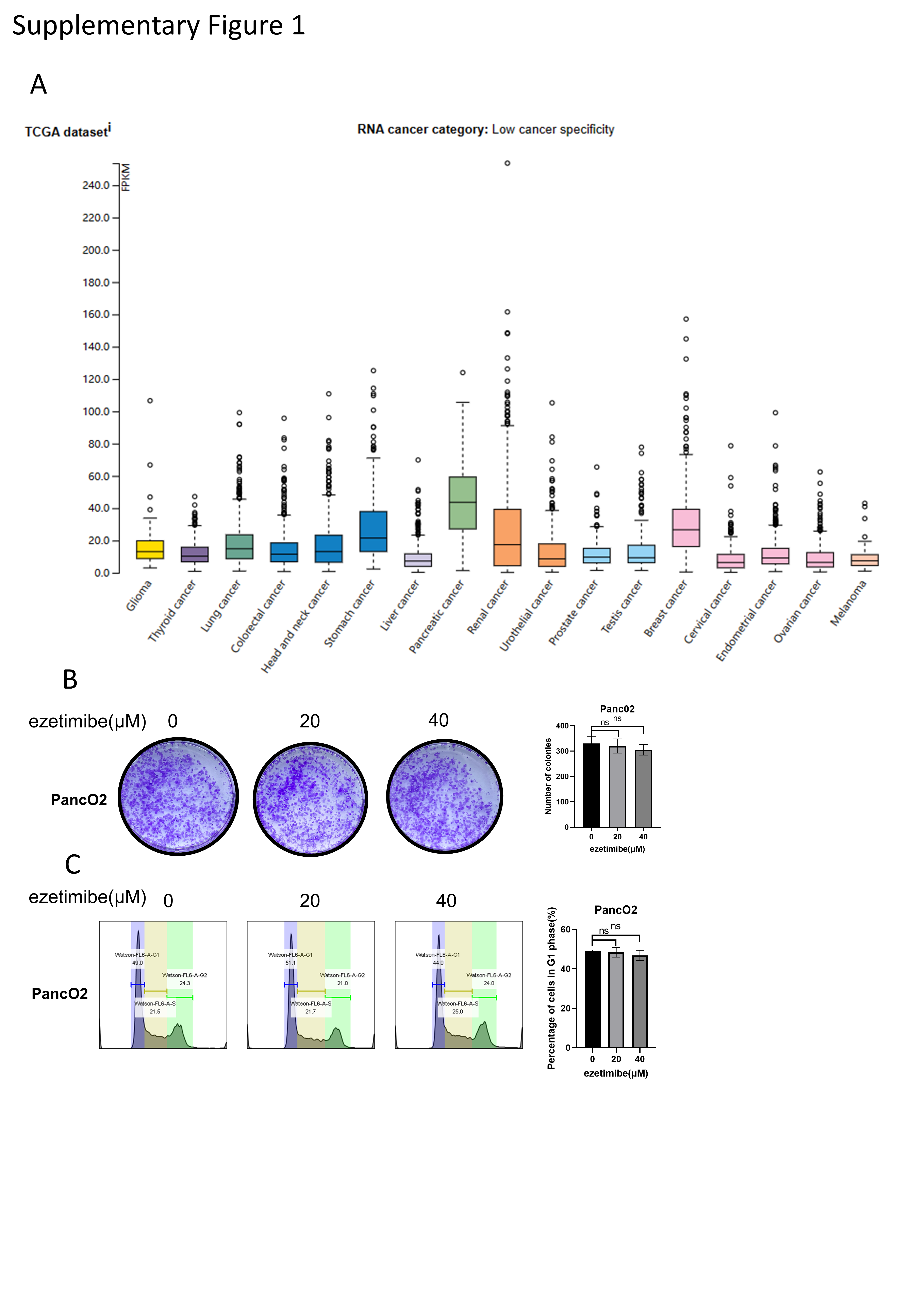


**Supplementary Figure1.ezetimibe does not affect the growth capacity and cell cycle progression of pancreatic cancer cells.**(A)According to the TCGA database, RNA levels of PDGFRB in different tumor tissues were analyzed.(B) Effects of different concentrations of ezetimibe on the clonal formation of pancreatic cancer cells PancO2. (C)Effects of different concentrations of ezetimibe on PancO2 cell cycle.
